# A Computational Clonal Analysis of the Developing Mouse Limb Bud

**DOI:** 10.1371/journal.pcbi.1001071

**Published:** 2011-02-10

**Authors:** Luciano Marcon, Carlos G. Arqués, Miguel S. Torres, James Sharpe

**Affiliations:** 1 EMBL-CRG Systems Biology Research Unit, Center for Genomic Regulation (CRG), Universitat Pompeu Fabra, Barcelona, Spain; 2 Departamento de Biología del Desarrollo Cardiovascular, Centro Nacional de Investigaciones Cardiovasculares (CNIC), Instituto de Salud Carlos III, Madrid, Spain; 3 ICREA Professor, Centre for Genomic Regulation (CRG), Universitat Pompeu Fabra, Barcelona, Spain; Princeton University, United States of America

## Abstract

A comprehensive spatio-temporal description of the tissue movements underlying organogenesis would be an extremely useful resource to developmental biology. Clonal analysis and fate mappings are popular experiments to study tissue movement during morphogenesis. Such experiments allow cell populations to be labeled at an early stage of development and to follow their spatial evolution over time. However, disentangling the cumulative effects of the multiple events responsible for the expansion of the labeled cell population is not always straightforward. To overcome this problem, we develop a novel computational method that combines accurate quantification of 2D limb bud morphologies and growth modeling to analyze mouse clonal data of early limb development. Firstly, we explore various tissue movements that match experimental limb bud shape changes. Secondly, by comparing computational clones with newly generated mouse clonal data we are able to choose and characterize the tissue movement map that better matches experimental data. Our computational analysis produces for the first time a two dimensional model of limb growth based on experimental data that can be used to better characterize limb tissue movement in space and time. The model shows that the distribution and shapes of clones can be described as a combination of anisotropic growth with isotropic cell mixing, without the need for lineage compartmentalization along the AP and PD axis. Lastly, we show that this comprehensive description can be used to reassess spatio-temporal gene regulations taking tissue movement into account and to investigate PD patterning hypothesis.

## Author Summary

A comprehensive mathematical description of the growth of an organ can be given by the velocity vectors defining the displacement of each tissue point in a fixed coordinate system plus a description of the degree of mixing between the cells. As an alternative to live imaging, a way to estimate the collection of such velocity vectors, known as velocity vector field, is to use cell-labeling experiments. However, this approach can be applied only when the labeled populations have been grown for small periods of time and the tensors of the velocity vector field can be estimated directly from the shape of the labeled population. Unfortunately, most of the available cell-labeling experiments of developmental systems have been generated considering a long clone expansion time that is more suitable for lineaging studies than for estimating velocity vector fields. In this study we present a new computational method that allows us to estimate the velocity vector field of limb tissue movement by using clonal data with long harvesting time and a sequence of experimental limb morphologies. The method results in the first realistic 2D model of limb outgrowth and establishes a powerful framework for numerical simulations of limb development.

## Introduction

The cellular processes by which a field of cells develops into a spatially-organized tissue have traditionally been split into two distinct questions: pattern formation and morphogenesis. The first focuses on the regulatory mechanisms underlying spatial and temporal cell fate specification. The second focuses on the cellular behaviors that physically drive growth and shaping of multicellular structures. While these two processes can indeed be considered to be conceptually separated, in practice they usually occur simultaneously and are believed to be tightly coordinated. The vertebrate limb is an excellent model system to study how these two processes work in combination [1]. In mouse, limb development starts around 9 days post fertilization with the protrusion of a mass of undifferentiated mesenchymal cells from the lateral plate mesoderm of the embryo. This structure, known as the limb bud, is able to grow and organize itself in less than 3 days to determine most of the structures found in the adult limb (tendons, skeleton, dermis etc.). Growth and patterning occur along three major axes: the proximal-distal axis (PD) that goes from the body to the finger tip; the anterior-posterior axis (AP) going from the thumb to the little finger and the dorsal-ventral axis (DV), going from the palm to the dorsal part of the limb. Important signaling centers of the limb, like the apical ectodermal ridge (AER) and the zone of polarizing activity (ZPA), are known to regulate both growth and patterning [2]–[4] and their activities are known to be highly coupled [5]. Moreover there is increasing evidence that tissue growth could play an important role for patterning [6]. Therefore a crucial step to better understand both limb morphogenesis and patterning is to accurately map tissue movements over space and time.

Several studies in chick [7]–[9] have produced fate maps which provide an important overview of the tissue movements in the mesenchyme and in the AER. In mouse, where in utero labeling is required, a first study was made in [10] by carbon particles injection and only more recently a first clonal analysis was performed by using a tamoxifen-inducible Cre reporter line to obtain cell labeling in the embryo [11]. However, the goal of these studies was not to build quantitatively-accurate maps of tissue movements, but rather to address specific questions about (a) the timing and mechanisms of regional fate determination, and (b) the possible presence of lineage-restriction compartments. It has been possible to draw clear conclusions regarding the second question: a clear compartment boundary restricting cells along the DV axis was found in [11], while no evidence for compartments along the other two axes was found (PD and AP). Indeed, both in chick and mouse a high degree of overlap was found between clones of the three PD regions corresponding to the stypolod, zeugopod and autopod. However, regarding the first question, experimental results have led to conflicting interpretations. For example, the relationship between PD patterning and the underlying tissue movement remains unclear. On one hand, limb tissue movement data has been used to support the idea that the three PD segments are specified at early stages of limb development [12] and subsequently only expand because of growth. On the other hand, comparisons between fate maps and the expression of distal markers like Hox genes [9], [13] have been used to support the idea that the PD patterning relies on complex spatio-temporal gene regulation coordinated with growth [14], [15].

Discrepancies between different interpretations of tissue movement data are due to a few specific limitations of previous studies which we aim to address through the modeling framework presented here. Firstly, quantitative details matter. Although alternative hypotheses may be qualitatively different from each other (such as the Progress Zone Model (PZM) [16] versus the Early Specification Model (ESM) [12]), the empirical evidence that could distinguish them will often depend on quantitative details of timing. Previous projects have revealed the overall pattern of movements, but have not mapped out the quantitative details. Secondly, all real data sets are sparse – they provide observations about a discrete collection of positions in space and time. It is non-trivial to extrapolate from these observations to a comprehensive prediction of how any piece of tissue will move at any point in time. Thirdly, many projects have employed a large time interval between labeling the cells and assessing the distribution of descendants. The shapes of the final labeled regions are therefore the result of an accumulated history of different tissue movements over time. Again, deconstructing the full sequence of local movements which lead to the final result is non-trivial. In particular, a recent study [17] showed that the tissue movements that drive limb morphogenesis are more complex than previously thought and depend on anisotropic forces. This and other studies [18], [19] also highlighted that limb mesenchymal cells have a complex shape and are capable of a wide range of cellular behaviors including oriented cell division and cell migration.

As an important step beyond the general overview given by previous fate maps, a formal numerical description of limb tissue movements over time would be of immense help to analyze the complex morphogenesis of the limb. Such a description could be defined by the velocity vectors defining the displacement of each tissue point in a fixed coordinate system. Ideally, the collection of these vectors, known as velocity vector field, would be directly measured by tracking tissue points during growth by time-lapse imaging. However, despite recent advances in live imaging of the mammalian limb bud [20], [21], it is a challenging technique and a complete description of tissue movements is still not available. In plants, clonal analysis has been explored as an alternative to live imaging for studying the growth of 2D leaves and petals. Local growth tensors were derived directly from shapes of clones and these data were combined into a computational model to recreate a full map of the global tissue movements (the velocity vector field) over time [22]. Unfortunately, in the mouse limb most of the available fate maps and clonal analyses have been performed with a long time-interval between labeling and analysis. This is ideal for the more common purpose of fate-mapping, as it reveals the final positions and tissue types of cells which were labeled during their early patterning phase. However such long-term clone shapes are not suitable for directly inferring local growth tensors – instead labeled populations should have undergone only enough growth to reveal local anisotropies, i.e. a short time-interval between labeling and analysis [23]. Another complication compared to the 2D plant case is the extensive mixing of mesenchymal cells that leads to a strong dispersal of the cells over space. A velocity vector field alone would therefore be insufficient to fully describe cell movements.

Due to this lack of quantitative data on tissue movements, most of the existing 2D mathematical models of limb growth have been used as theoretical tools to explore possible cellular hypothesis explaining limb outgrowth [24], [25]. These studies relied on the existing literature to suggest the underlying mechanics of limb growth, and consequently predicted tissue movement maps consistent with the proliferation gradient hypothesis [26], in which tissue expansion is concentrated at the distal tip. However, a recent simulation which performed a more rigorous comparison of this hypothesis against quantitative data on proliferation rates, has demonstrated the implausibility of this concept [17]. Since it is now clear that the cellular mechanics underlying outgrowth are complex and not well understood [19], [27], we therefore wished to create a model of tissue movements which is based not on a mechanistic hypothesis, but which instead acts as a numerically-descriptive framework within which to integrate information from real clonal experiments. In particular we have developed a methodology which is not restricted to short-term clonal data, and which can interpret the available long-term clonal and predict cell movements across the full spatio-temporal extent of mouse limb bud development. The model is able to use 2 sources of data as empirical constraints to define a biologically-accurate tissue movement map: (i) a temporal sequence of experimental limb bud morphologies – a numerical shape description for every hour of development over a 72 hour period, and (ii) a collection of clonal data generated by a tamoxifen-inducible Cre transgenic mouse line.

The paper is organized as follows. In the first two sections of the results we introduce the new computational approach that was developed to explore different limb tissue movement maps matching experimental change in limb morphology. In the subsequent two sections we present a mouse clonal analysis of early limb development (from 9 to 12 days pf.) and we show how the tissue movement map that better matched the distribution and shapes of clones was selected. In the following section, the tissue movement map is modified to match the experimental degree of cell mixing observed in the experimental clones. In the last section of the results we characterize the tissue movement map and relate it to traditional PD patterning hypothesis. Finally in the Discussion section we summarize the study and present the future applications of the model.

## Results

We present here a new computational method that estimates the velocity vector field of limb tissue movement by using 2 experimental constraints: a sequence of experimental limb morphologies, and long-term clonal data. The main idea underlying the method is to generate a set of hypothetical velocity vector fields that are consistent with the first constraint (the experimental morphological changes), and then to select the one responsible for real limb outgrowth by comparing simulated fate maps with the second constraint (the experimental clonal data). In this respect our approach is analogous to a “reverse-engineering' method, as the data cannot lead to a direct calculation of tissue movements but can only constrain the possible forward simulations. The resulting velocity vector field was then used to derive the tensors of the velocity gradient which describe the local behaviors of the tissue movement. As an application of the model, a reverse version of the tissue movement map was generated to provide a relative estimate of the degree of mixing between the progenitors of three PD segments. Finally, by mapping a time course of Hoxd13 gene expression into the model, we were able reveal the contribution of tissue movement to the expansion of the Hoxd13 domain.

### From 2D experimental morphologies to velocity vector fields

**Figure 1:**
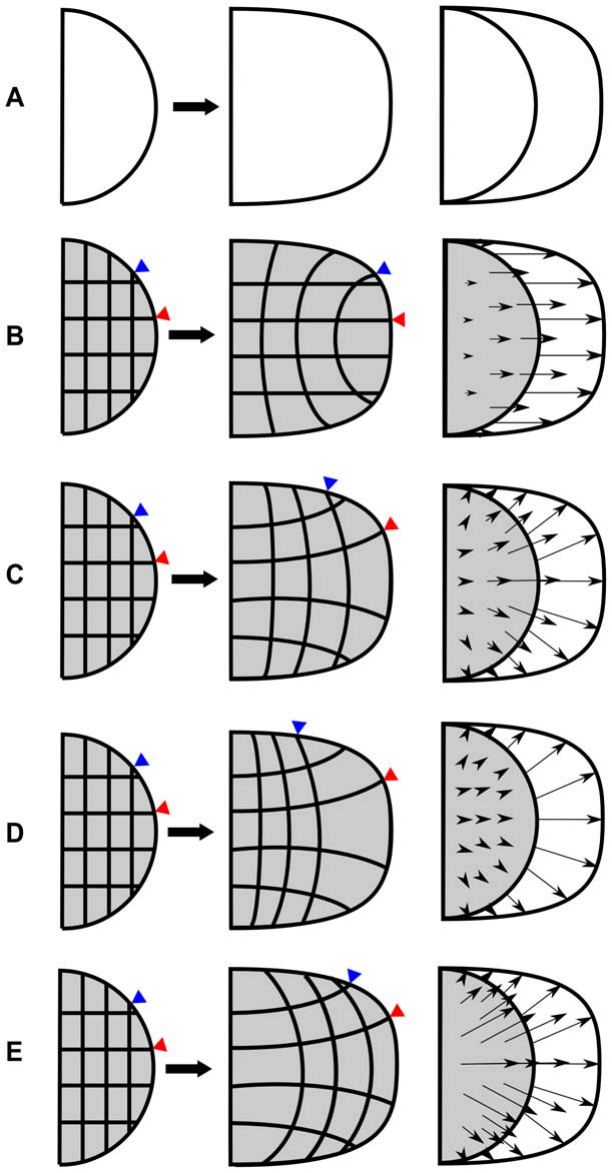
Different ways to make the same shape. (A) A simple semi circle (first column) grows into a defined shape (second column). The two shapes are aligned at their left boundary (third column). (B) A velocity vector field pointing in the distal direction with a vector magnitude distribution that leads to a uniform expansion. (C–E) A variety of velocity vector fields which can all create the same boundary shape change. The first magnitude distribution (C) defines uniform expansion, the second (D) defines a greater distal expansion and the third (E) a greater proximal expansion.

The shape of the limb bud at any point in time can be defined by a spline curve, but clear morphological features along this line are absent – the limb displays a smooth rounded shape. This lack of landmarks means that even a very precise knowledge of the shapes over time is insufficient to define the underlying tissue movements. This is true both for the internal tissue and also the limb boundary itself, as a particular point of tissue (or landmark) could slide along the boundary spline without altering the shape. Figure 1 illustrates the nature of the problem: a well-defined shape change is given (panel A), but numerous different tissue movement maps are all equally compatible with this observation, each of which has different combinations of local tissue speed and directionality (B–E). Despite these degrees-of-freedom, the temporal sequence of boundary shapes does nevertheless act as an important constraint on the full range of growth possibilities. To capture an accurate numerical description of these shape changes we therefore took advantage of a morphometric analysis performed in [28]. In that work, 600 limb buds of different ages were photographed in a standard orientation, cubic splines were fitted to the boundaries, average shapes were calculated for key timepoints, and shape interpolation was performed to calculate the intermediate shapes [28]. The result is an hour-by-hour sequence of 72 shapes which represents a standard trajectory of limb bud morphology over developmental time (from approximately E9 to E12) – see Figure S1. Each shape corresponds to a morphometric stage that is named as mE*dd*∶*hh*, where *dd* is the morphometric embryonic day and *hh* is the morphometric embryonic hour. It is important to note that the morphometric stage notation is different from the standard embryonic day notation, for example the standard notation E10.5 translates into the morphometric stage mE10∶12.

**Figure 2:**
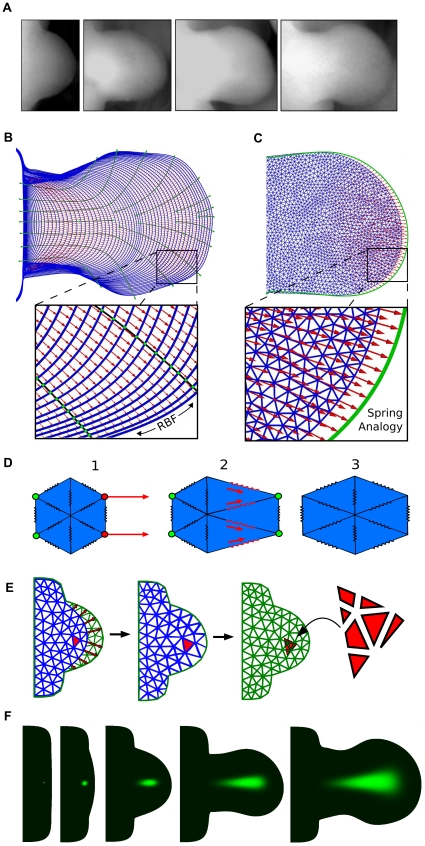
From morphologies to velocity vector fields. (A) A sequence of limb photos at different developmental stages. (B)The chronological sequence of limb morphologies derived from [28] (blue color) is overlaid by the *boundary control splines* (black lines). Intersection points between control splines and limb morphologies (green dots) define a set of control vectors that are interpolated using radial basis functions (RBF) onto the boundary mesh points. In this way a series of velocity vector fields is obtained (red arrows) which define how to displace the boundary mesh points to match the following mesh in the sequence. (C) Starting from the boundary displacement, a velocity vector field that displaces internal mesh points (red arrows) is calculated by using an edge spring analogy. (D) An example of deformation obtained with an edge spring analogy. 1) A deformation is applied to the boundary points of the mesh (red points) and the displacements of mesh points of the left-most boundary are fixed to zero (green points). 2) Edge springs of the triangles close to the deformation exercise tension forces to the neighboring triangles. 3) Relaxation of the forces to reach equilibrium provides a smooth deformation of the mesh. (E) A mesh (blue mesh) is deformed to match the next mesh in the sequence (green mesh). A triangle (red triangle) of the deformed mesh is split into the overlapping triangles of the next mesh by considering the respective areas of overlap (seven red segments on the right). (F) An example of virtual fate map. A triangular element is labeled with a green dye (probability equal to one) at early stages of development and its fate is simulated using the sequence of deformations and interpolations.

For this study we had to develop software that would allow the exploration of a set of possible velocity vector fields that can each reproduce the same observed boundary changes. In particular, different tissue movement maps are equivalent to considering the 2D limb shape as a rubber sheet or mesh, with different distributions of elastic deformation (e.g.. the various mesh deformations shown in Figure 1B–E). To cover the whole temporal sequence of development, a single complete map would include a sequence of slightly changing hour-by-hour deformations across all 72 shapes. We thus spatially discretized each of the limb bud shapes using an unstructured triangular grid. An example of the type of grid generated is given in Figure 2C. We then sought a convenient approach to parameterize the variety of possible mesh deformations across time, and devised a two-step method – the first step dealing with boundary, and the second with the internal tissue movements.

For the first step, in effect we must define a series of landmarks which explicitly map points in each boundary shape to their positions in the next shape (equivalent to controlling the blue and red triangles in Figure 1). To maximize the flexibility of the system we devised the concept of user-defined *boundary control splines*. A graphical user interface was developed to allow arbitrary positioning of these control splines onto the sequence of limb shapes (an example is shown in Figure 2B). Intersections between control splines and limb shapes (the green points in Figure 2B) determined, for each pair of contiguous limbs, a set of control vectors which defined how to displace points on a young limb shape in order to match a point on the next older one. Due to the intrinsic smooth curvature of these splines over space, they are a convenient method for defining smooth boundary movements over time (Figure 2B). Since the resulting control vectors were defined for arbitrary points of the limb boundaries they were interpolated onto all the mesh boundary points using radial basis functions (RBFs) with Gaussian basis. For each limb mesh, the 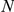 control vectors 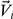, 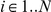 (calculated from the intersection between the spline curves the limb morphologies) were used to derive two radial basis function interpolations 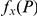 and 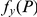 with the formula:
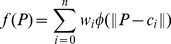
(1)where the Gaussian basis 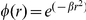 and 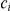 is the origin of the control vector 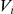. Coefficients 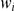 were estimated using the matrix methods of linear least squares to fit the 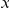 components of the control vectors 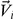 in the case of 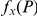 and the 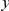 components of the control vectors 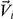 in the case of 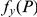. Interpolated velocity vectors 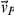 were thus calculated as 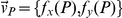 for all the points on the mesh boundaries. This results in a series of velocity vector fields (the red arrows in Figure 2B) which define how to displace the boundary points of each limb mesh in order to match the following in the chronological sequence. We have thus created a method by which the user can conveniently define a variety of different mappings for the boundary, which are all consistent with the experimental shape changes.

For the second step we had to devise a way of defining the internal tissue movements (the velocity vector field), and in particular a method for exploring some variations on these maps. The internal movements must be consistent with a given boundary mapping (defined above) and we therefore chose to calculate internal velocity vectors using an edge spring analogy algorithm which can smoothly propagate a given set of displacements from the boundary into the internal points of each mesh. Spring analogy algorithms are a popular approach to deform mesh elements by modeling edges as lineal tension springs [29], Figure 2D. Although this approach is sometimes used to model the elastic mechanics of a tissue, in our case we are not assuming that it correctly represents the physical properties of the tissue – it is simply an efficient method for defining hypothetical displacement maps. The algorithm prevents node element collisions and for small deformations it ensures that the quality of mesh elements is maintained. In our model this approach exhibits particularly good performance due to the small time differences between contiguous limb morphologies (differing by just one hour of development). Moreover the smooth propagation of displacements translates into a smooth spatial distribution of tissue expansion in accord with the limb proliferation maps presented in [17]. Furthermore, since each spring in the mesh can be given a stiffness coefficient, it also presents a convenient method to explore variations to the tissue movement map, simply by varying the spatial distribution of the stiffness (explained in more detail below).

Our spring analogy method was implemented as follows: given a mesh 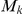 defined as 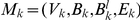, where 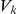 are the mesh points, 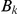 the points on the mesh boundary, 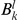 the points on the left-most boundary and 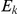 the edges of the mesh, our algorithm proceeds as follows:

1. the displacement of all the boundary points on the left-most boundary are set to zero (representing the deep internal tissue of the body) and the displacements of the remaining mesh boundary points is set to the velocity vectors calculated with the radial basis function interpolation described above in equation (1):
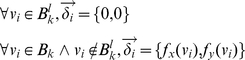
(2)where 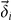 is the displacement vector of the vertex 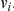

2. The following iterative formula is used to equilibrate the forces 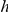 times until the displacements are close to zero, (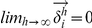):
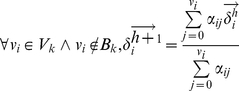
(3)where 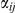 is the stiffness coefficient of the edge 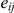 that connects the point 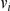 and 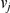 of the mesh.
3. Eventually the new vertex positions are calculated as:
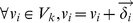
(4)A small graphical example of the algorithm is shown in Figure 2D.

A variety of alternative movement maps which all fit to the given boundary displacements, can now easily be generated by altering the spatial distribution of the stiffness coefficient 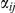.

In conclusion, we can define a velocity vector field for each mesh in the chronological sequence (the red arrows in Figure 2C) representing a hypothetical tissue movement map connecting each pair of contiguous morphologies in the sequence, see Video S1. The displacements on the boundary can be altered through the use of the boundary control splines, while the internal displacements can be altered through changes in the spatial distribution of spring stiffness. The collection of 9 different maps explored extensively in this paper are described later.

### Triangle interpolation map and virtual fate maps

In this section we wish to generate virtual clone experiments, which can later be compared to real clonal data. Although the velocity vector fields defined above are smooth across time, to create virtual clones the resulting hour-by-hour mesh deformations must be linked together to allow tracking the fates of individual tissue regions over time.

Each of the 72 velocity vector fields defines how to deform a limb mesh in order to match the following mesh in the sequence, and the complete set of fields describes a hypothetical computational tissue movement map that matches the entire sequence of experimental morphologies. To track a region of tissue over the full 72 hours, we must determine how the triangular elements of each mesh will map to the different set of triangles of the 1-hour older mesh. In particular, we must calculate a triangle-interpolation map from mesh to mesh. A graphical representation of this process is provided in Figure 2E. On the left of this panel, a blue mesh is deformed according to its velocity vector field (red arrows), in the second column of the figure, the deformed blue mesh is perfectly overlapping the next green mesh in the sequence. On the right of the panel, a triangle of the blue mesh (red labeled triangle) is split in 7 parts. Each of these parts represents the area of overlap between the original triangle of the blue mesh and a triangle of the next green mesh. In the course of a numerical simulation, numerical values associated with the triangle of the blue mesh are transferred to the triangles of the next green mesh according to the area of overlap.

Repeating this operation for each pair of contiguous meshes, we generate a correspondence map that defines the fate of each triangle of the first mesh in the sequence (stage E9) with respect to a set of triangles on the last mesh in the sequence (stage E12). This map is a computational implementation of an experimental fate map. The interpolation is conservative and is based upon the velocity vector fields that define the whole virtual tissue movement. A virtual fate map can be performed by marking a triangle with a “virtual clonal dye” – in practice by assigning the triangle a probability of one and then following the evolution of the probability distribution over time, see Figure 2F and Video S2. Experimental clones can be seen as a stochastic simulation of these probability distributions. (Further details on the use and meaning of this map are provided in Text S3).

This approach has also a more general application, since it defines the basis for any kind of numerical simulation on a growing triangular mesh representing limb growth. Indeed, by interpolating numerical values on a newly generated mesh at every hour of development we are effectively implementing a global re-meshing scheme that avoids the large element deformations that a grid would undergo over the whole 72 hours of limb development. It is well known that frequent re-meshing introduces a source of spatial diffusion in numerical solutions [30], [31]. This is not however a problem in the context of virtual fate maps of the limb bud – clonal data of mouse and chick show a high degree of cell mixing even for short time intervals after clone induction. In the virtual fate maps, the probability distribution represents the local density of labeled cells, with a value of one corresponding to a region where every cell is labeled. The local decrease of labeled cell density which is modeled as diffusion therefore corresponds to cell mixing. Determining the appropriate levels of cell diffusion/mixing for the model is discussed in a later section.

### Mouse clonal data

In this section we present a mouse clonal analysis of early hind limb development, which will subsequently be used to compare with hypothetical virtual clones. These experimental results are compared with previously published fate maps in chick and implications on PD patterning are discussed.

**Figure 3:**
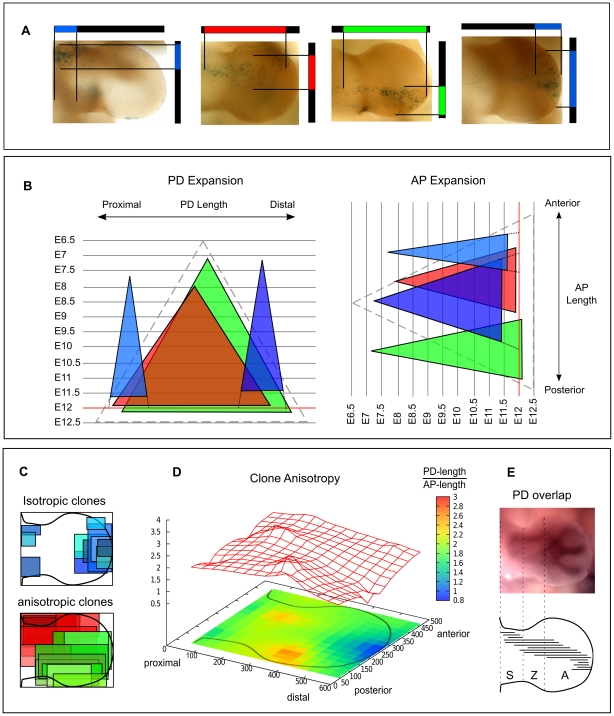
Clonal analysis. (A) Four clones showing the quantification of the AP and PD clone lengths. (B) In order to compensate for the variation in developmental stage between different embryos each limb was staged and the tamoxifen injection time was adjusted accordingly. Large triangles represent the AP and PD clone expansion over space and time. PD and AP lengths were mapped at E12 (red line) considering prospective (dotted line) or retrospective lengths. (C) Each rectangle represents the AP and PD length of one clone. Clones were clustered into two groups: isotropically expanding clones, with comparable AP and PD length (blue rectangles), and an-isotropically expanding clones having the PD length greater than AP length (red and green rectangles). (D) A graph showing the degree of clone anisotropy in the limb, PD length over the AP length. Blue means low anisotropy and red high anisotropy. (E) Top: In-situ of Sox9, a known early skeletal marker showing the position of the three PD segments (S = stylopod, Z = zeugopod, A = autopod) Bottom: 16 clones showing the degree of overlap between clones spanning across different PD segments.

We used the tamoxifen inducible Cre-line presented in [11] to conduct a mouse clonal analysis from stage E9 to stage E12 of development. The clones were induced by injecting low tamoxifen concentration at E8 (0.10mg) so that random recombination events would produce single cell labeling events within the embryos. 24 hind-limbs showing suitable monoclonal labeling were used for the clonal analysis. To compensate for the variation in development between embryos of the same litter and the uncertainty of the injection day, we staged each limb using the staging system presented in [28] and adjusted the estimation of the injection day accordingly. The PD and AP clone lengths relative to the maximum PD and AP length of the limb were measured as shown in Figure 3A (See also Figure S2). From the quantification of clone lengths, two graphs representing respectively PD and AP clone expansion were produced, an example is shown in Figure 3B. All clone lengths were mapped at stage E12 by considering prospective or retrospective lengths as shown in Figure 3B. PD and AP lengths at E12 were visualized representing each clone as a rectangle centered in its AP and PD midpoint (in Figure 3C). In this way we were able to cluster the clones in two groups according to their position and shape: a) isotropically expanding clones in the proximal and distal part of the limb that showed similar AP and PD expansion rate (highlighted in blue), b) anisotropic clones that expanded more along the PD axis than the AP axis (highlighted in green and red). Plotting the ratio between PD and AP lengths a similar behavior was revealed (Figure 3D). In accord with a previous study [11] we found no clear evidence for AP and PD compartments. Indeed, a high degree of cell mixing was observed across the whole limb. Consistently with previous studies in chick [8], [9] we found that clones expanded across one or two PD segments but never span across the whole PD axis of the limb, see Figure 3E. Remarkably, no clones were found restricted to the zeugopod alone – all clones found in this zone also overlapped with the autopod or the stylopod regions.

### Tissue movement map estimation

Now that we have (i) a method for generating virtual clones on hypothetical growth maps, and (ii) a suitable set of experimental clone data, the next task is to use the latter to infer a biologically-realistic growth map for the mouse limb bud. This task can be split into two parts: firstly defining a suitable set of hypothetical velocity maps, and secondly developing a method to systematically compare each map against the experimental clone data.

The space of all possible movement maps is highly multidimensional and potentially very large, (as exemplified in Figure 1). Exhaustively exploring all theoretically-possible maps would have a prohibitive computational cost, and the challenge is therefore to find a good match in an efficient manner. However, when experimentally-derived assumptions about limb development are considered – for example that tissue never moves backwards towards the body, and that the distribution of tissue growth varies smoothly over space – in fact the available options reduce to a basic set of possible asymmetries, as summarized by the following questions: Is there asymmetric growth along the AP axis? E.g. does the posterior tissue expand faster or slower than the anterior tissue? Similarly, does the distal region expand faster or slower than the proximal region? Finally, does the tissue grow fairly straight distally, or alternatively does it fan-out along the AP axis into the autopod?

**Figure 4:**
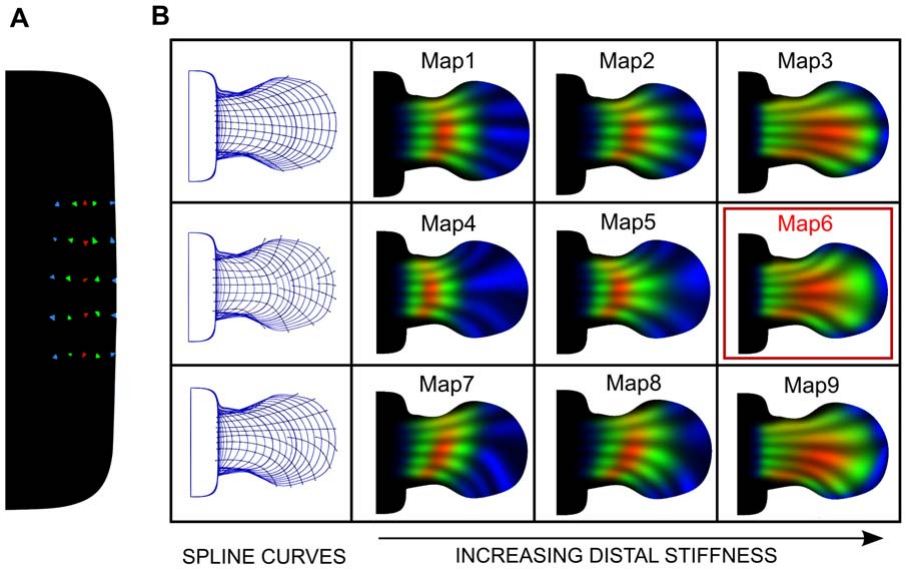
A collection of tissue movement maps. (A) Initial conditions used for the comparison between the tissue movement maps. Clones are positioned on a grid along the AP and PD axis and are colored according to the PD position, from proximal to distal: blue, green,red, green and blue. (B) Virtual fate maps resulting from 9 different maps obtained combining different stiffness coefficient distributions and spline curves (described in more detail in the main text). The left column shows the control spline curves. The stiffness of the distal springs is increasing from left to right. The tissue movement map outlined in red (Map6) is the one that best matched the mouse clonal data.

Using the two levels of manual control described in a previous section of results (the boundary control splines, and the spring stiffness distribution) we were able to create a collection of 9 maps which represent the main plausible asymmetries in limb bud development (Figure 4). Since the control splines are oriented substantially along the PD axis, varying their positions primarily affects the AP distributions of growth. We were thus able to choose 3 sets of these control splines, which define either a fairly straight distally-oriented growth (Maps 1–3), a strong fanning-out movement into the distal autopod region (Maps 4–6), or a posteriorly-biased map in which growth is preferentially twisted into the posterior region (Maps 7–9). Using the second level of control – the spring stiffness distribution – we could vary the PD growth pattern. The stiffness coefficient for each edge of the mesh was given a spatial distribution in the following way:
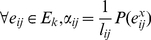
(5)where 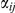 is the stiffness coefficient of the edge 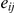 that connects the point 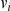 and 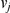 of the mesh, 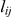 is the length of the edge 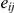 and 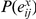 is a scaling function used to vary the distribution of the stiffness coefficients according to 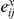, the 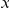 coordinate of the edge.

We explored a number of scaling functions, 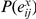 in equation (5), to vary the stiffness coefficients along the PD axis (the x axis), and chose 3 which display different degrees of bias towards the distal end of the limb. The first function used was an inverted sigmoid (with respect to x) that defined lower stiffness coefficients in the distal part of the limb (Maps 1, 4, 7), i.e. allowing greater expansion of distal tissue. The second function was a constant value defining no change in stiffness along the PD axis (Maps 2, 5, 8). The third function was a sigmoid that defined higher stiffness coefficients in the distal part of the meshes (Maps 3, 6, 9), i.e.. restricting distal growth along the PD axis. The combination of the 3 AP variations, and the 3 PD variations resulted in 9 maps to be explored in detail. Additional details regarding the maps and the scaling functions are provided in Text S1. As an important control, proliferation patterns were derived from the velocity vector gradients of each map and the ranges were ensure to be biologically realistic, see Figure S3.

The second step was to evaluate which of the 9 hypothetical tissue movement maps best fitted the experimental data. We mapped the 13 clone pictures having better contrast and best capturing the main features of the clonal data set onto the last triangular mesh in the sequence (stage E12). This was done by manually aligning the limb morphologies of the thresholded clone pictures on the last mesh boundary. Results are shown in Figure S4. Next we implemented an algorithm to systematically compare all possible virtual clones for a given map with the 13 experimental clones. The youngest timepoint (E9) is represented by a mesh with 3156 triangles, and so this is also the number of virtual clones which could be calculated for each of the 9 maps. Evaluating the score of a given map therefore involved over 40 thousand clonal comparisons, which were calculated in the following way:

Given a set of triangles representing a virtual clone 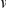 and a set of triangles representing an experimental clone 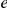, each virtual clone was scored with the formula:
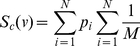
(6)where 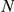 is the number of common triangles between 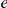 and the virtual clone 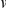, 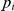 is the probability value associated with the triangle 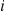 of the virtual clone and 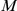 is the number of triangles of the experimental clone 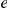. The first part of the scoring function represents the probability that the experimental clone is obtained from the spatial probability distribution of the virtual clone. The second part acts as a penalty for cells founds outside the domain of the virtual clone. It calculates a score between 0 and 1 describing the proportion of triangles of the experimental clone contained in the virtual clone.

**Figure 5:**
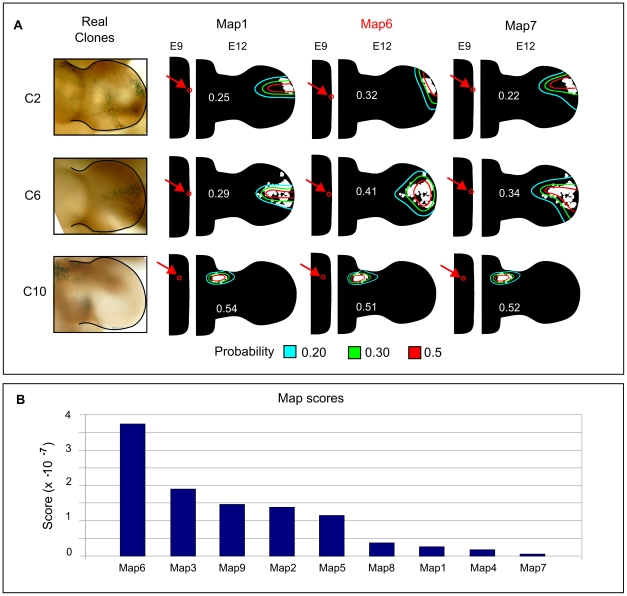
Virtual clone scores. (A) The left column shows pictures of experimental clones. The remaining columns show numerical comparisons between the experimental clones (white shapes) and the best matching virtual clone (colored contour lines). The comparison is made for three different maps (Map1, Map6, Map7). The contour lines define three regions of probability of the virtual clones: the area enclosed by the red line contain the 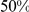 of the clone probability, the area between the green and the red contour the 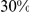 and the area between the blue and the green contour the 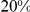. The number in white is the score value for each virtual clone. Limb shapes at stage E9 show the initial triangle associated with the virtual clone. (B) A comparison between the total scores of the 9 virtual tissue movement maps. Map6 scores almost 2-fold better than all the other maps.

Figure 5A shows the positions that scored the best for three experimental clones on three different maps. Experimental clones are shown in white while virtual clones are visualized with colored contour lines that define three regions of probability: the area enclosed by the red line contains the 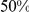 of the clone probability, the area between the green and the red contour the 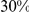 and the area between the blue and the green contour the 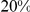. Detailed clone scores for map are shown in Figure S5. The total score for each map was calculated by using the formula:
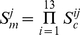
(7)where 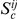 is the best score found for the clone 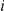 on the map 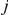 using the formula (6). We calculated the product between the clone scores (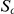) in order to give higher score to the maps that better matched all the experimental clones. Figure 5B shows the total score for each map. In conclusion, Map6 scored almost two-fold better than the other maps and was therefore selected as the one the best represented hind-limb tissue movement. As a test of robustness of this result, we chose to remodel the tissue movements of Map6, but on a finer mesh (starting with 5678 triangles at E9, instead of the previous 3156). These results (shown in Text S2) highlight that the same positions and orientations of virtual clones were obtained irrespective of the mesh resolution.

### Cell mixing estimation

**Figure 6:**
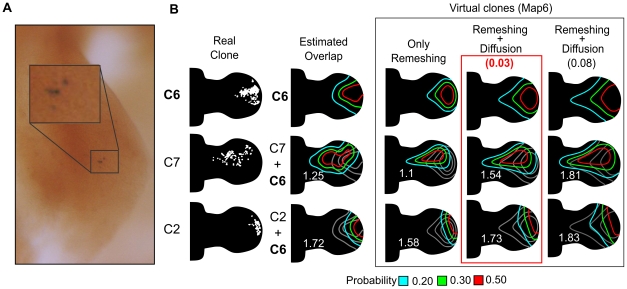
Re-meshing and cell mixing. (A) A picture showing the degree of cell mixing observed at early times after clone induction. (B) The first column shows 3 experimental clones used for this analysis. The second column shows the estimated probability distributions of experimental clones obtained by a mean filter. The second and the third row of this column show a quantification of the overlap between pairs of experimental clone distributions – C6-C7 and C6-C2. The number in white is the score representing the amount of overlap. In the three right-hand columns the overlap between the correspondent virtual clones from Map6 is calculated by considering different amount of additional diffusion: no additional diffusion (first column), a diffusion constant of 0.03 (second column) and a diffusion constant of 0.08 (third column). It can be seen that addition of some diffusion improves the score (compare with the “Estimated overlap” column), while too much extra diffusion makes the scores worse again.

As mentioned in the previous sections, the hourly global re-meshing process introduces an inherent source of diffusion to the numerical simulation, which we consider equivalent to the redistribution in the density of labeled cells. This is in agreement with our clonal data that clearly shows a decrease of labeled cell density during early phases of clone expansion resulting from the mixing between labeled and non-labeled mesenchymal cells, see Figure 6A. An accurate quantification of the degree of mixing between mesenchymal cells would require a larger collection of clonal data, but we nevertheless wished to provide a rough estimate of the experimental degree of cell mixing and compare it with the amount of cell mixing introduced by the re-meshing process. Taking advantage of our model, we decided to estimate cell mixing by quantifying the degree of overlap between the experimental clones. The quantification was performed in two steps.

First, we estimated the spatial probability distribution of three different clones. This was done by applying a mean filter to the experimental clones as they were mapped into the mesh at stage E12. The mean filter averaged the value of each triangle with its direct neighbors and normalized the overall spatial distribution to 1. By iteratively applying the filter we smooth the distribution of the labeled triangles until the data did not present any spatial discontinuities. The estimated probability distributions of three experimental clones is presented in the second column of Figure 6B.

Secondly, we defined a score to quantify the degree of overlap between clone probabilities distributions. Given two clones 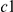 and 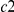, the score was defined as:
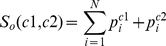
(8)where 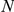 is the number of common triangles between the two clones, 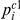 is the probability value associated with the triangle 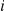 of the clone 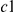 and 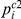 is the probability value associated with the triangle 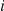 of the clone 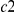. The overlap between two pairs of experimental clones was quantified using this formula (Figure 6B, second column). Results were then compared with the quantifications of the overlap between the correspondent virtual clones of Map6, see the third column in Figure 6B. Our estimations of overlap suggested that the amount of cell mixing introduced by the global re-meshing process was not enough to mimic the real degree of clone overlap consistent with the experimental data.

We therefore introduced an additional diffusion term that would model a higher degree of cell mixing. The quantification of the clone overlap was repeated multiple times with different degrees of extra-diffusion. In this way we were able to provide a rough estimate of the diffusion constant that better fitted the experimental clone overlap (see the two columns on the right in Figure 6B), and importantly to show that the unavoidable diffusion introduced by our global re-meshing scheme must in fact be augmented with extra diffusion to reach biologically-realistic levels.

**Figure 7:**
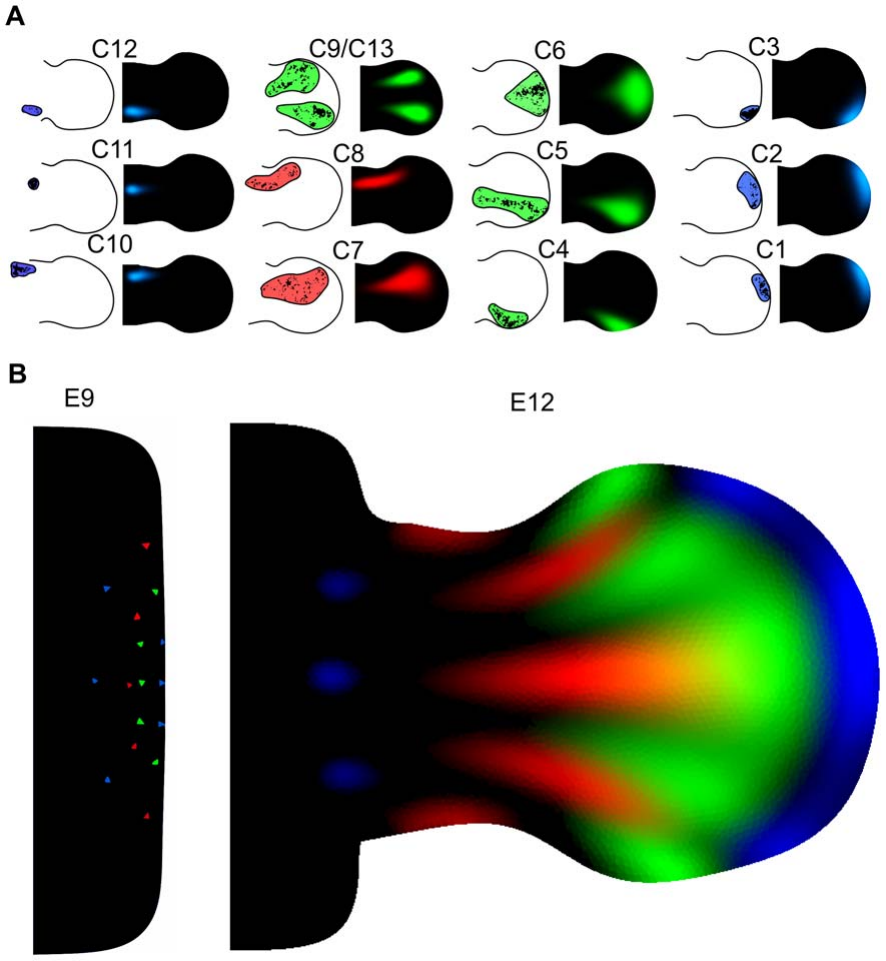
The matching map. (A) A direct comparison between experimental clones (white background) and simulated clones (black background). Experimental clones have been thresholded and the clone shape has been highlighted. (B) A collection of clones matching the distribution and shape of the experimental clonal data. Blue clones expand isotropically on the PD and the AP axis, and green and red clones expand more on the PD axis. On the left, the initial conditions of the fates are shown.

In conclusion, the refined version of Map6 with the extra-diffusion not only matched the shape and distribution of the clonal data but was also matched the relative positing and spatial extension of the clones. A qualitative comparison between the experimental and the virtual clones obtained with this map is shown in Figure 7A. A simulation showing a number of clones that match the distribution and shape of the experimental clonal data is shown in Figure 7B and Video S3.

### Applications of the model

In this section we present some applications of the growth model that highlight the power of mathematical modeling in characterizing limb outgrowth in space and time.

**Figure 8:**
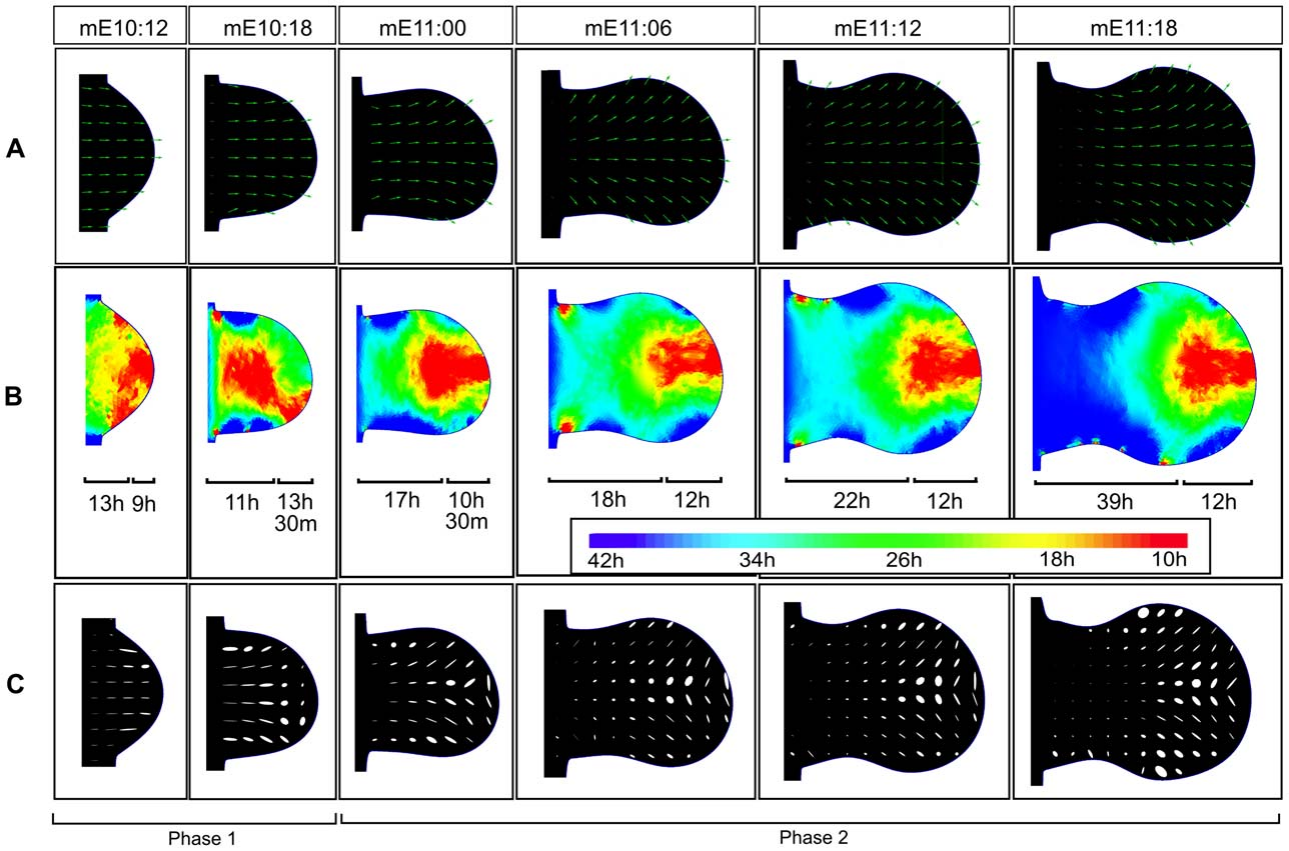
Derivation of growth tensors. (A) The velocity vector field of the map that best recapitulates limb tissue movement. Velocities have been normalized for clarity. (B) A heat map visualizing the expansion rate of each triangle. Red corresponds to high expansion rate (low cell cycle time of 10h) and blue to low expansion rate (high cell cycle 42h). Average cell cycle times of distal (1/3 of the PD axis from the tip) and proximal parts (remaining 2/3 of the PD axis) are shown for each time point. (C) Ellipses visualizing the anisotropy and rotation of different parts of the tissue. During an initial phase of development the anisotropy is relatively uniform (until stage mE10.18) and oriented towards the distal tip of the limb. After this stage the anisotropy is non-uniformly distributed, and is higher in the region under the influence of the AER. In the central sub-ridge region the direction of the anisotropy is parallel to the AER, while in more anterior or posterior regions it is perpendicular to the AER.

A first interesting application of the model was to derive and visualize the local tissue behaviors that contributed to the global tissue movement responsible for limb outgrowth. In the model, local tissue behaviors can be represented by the growth tensors associated with each mesh triangle. Tensors were derived from the spatial gradient of the velocity vector field and provided three useful pieces of information: tissue growth rate, anisotropy and rotation [32]. The first of these can be directly related to proliferation and we translated these values into cell cycle time by considering the time required to double the area of a triangle. A similar approach was taken in [17] by assuming that the cell cycle time was equivalent to the time required to double the volume of a tetrahedron in a 3D limb tetrahedral mesh. Tensors were calculated for each time point and expansion rates were visualized using heat maps, see Figure 8B. Our model predicted a proliferation distribution with shorter cell cycle times in the distal region, with an average value of 9h, and longer cell cycle times on the proximal part of the limb, with an average value of 24h. The difference between the two regions was more evident from the stage E11 onwards with an average maximum difference of 12h in agreement with published experimental proliferation maps [17]. The other two components of the tensor were visualized using ellipsoids that were scaled and rotated according to the anisotropy and the rotation, see Figure 8C. The model predicted an initial relatively uniform anisotropy (until stage mE10.18) that was oriented towards the distal tip of the limb. This reflected the initial phase of elongation and protrusion of the limb and confirmed the results presented in [17] that showed that the elongation of the limb bud cannot depend only on differential isotropic cell proliferation but has to depend on anisotropic tissue movement. The model also revealed a second late phase, after mE10.18, in which the anisotropy under the distal ectoderm close to the AER was higher than in the central tissue. Interestingly, within the most distal/central region of this sub-ridge mesenchyme the direction of anisotropy was parallel to the AER, whereas it was perpendicular to the AER in more anterior and posterior regions. Taken together these results suggested the possibility that during autopod expansion, signals coming from the distal AER could act promoting a higher proliferation rate and an anisotropic behavior of the cells that result in the expansion of the autopod along the AP axis.

The second application of the model focused on the PD patterning of the limb. In particular we used our model to address a matter of debate for the last two decades: that is to identify at which stage the three PD segments of the limb can be specified. The problem has been addressed numerous times in the chick by creating fate maps to study two different aspects: the degree of mixing between cells of the prospective segments, and to follow the lineage of cells expressing markers of the three PD segments, like Hoxa13 and Hoxd13 for the autopod. An early study using both approaches [7] concluded that the degree of mixing between the prospective PD segments was low and that the anterior expansion of the distal marker Hoxa13 was mainly due to growth. In other words this study suggested that the distal segment was specified early during development and that it subsequently expanded due to growth. A similar idea was further developed in [12] where it was proposed, based on the observation that early fate maps were almost always confine to a single segment, that the three segments were specified early in development and that their expansion was again mainly due to limb growth. This idea became known as the Early Specification Model. In contrast, a more recent study in chick [9] concluded that the mixing between zeugopod and autopod was higher than that between stylopod and zeugopod at early stages. This together with analysis of Hoxa13 and Hoxa11 expressions suggested that the two more proximal segments were specified early while the distal segment was specified later during development. However, another study in chick [8] found no strict barriers between all the three segments at early stages. Recent results in mouse [13] also supported a similar view by showing that cells expressing known markers of three segments were capable of altering their expression to match the local environments where they were moved. This supported the idea proposed in [14], [15] that identities were progressively specified over time by active regulation along the entire limb despite cell transit between the segments.

Considering some of the controversies mentioned above we decided to use our model in two ways: firstly to give an estimation of the degree of mixing along the PD axis, and secondly to estimate the extent to which limb tissue movement could be responsible for the expansion of the Hoxd13 domain, one of the known distal marker of the autopod.

For the first question we used the model to compute a reverse version of the tissue movement map. This would allow us to start by marking the three PD segments at the oldest timepoint (E12), and then work backwards to determine which regions of the young limb bud could contribute to the 3 segments. Importantly, this is not equivalent to running a clonal experiment backwards in time. As in a traditional heat diffusion problem, individual virtual clones cannot be reverse simulated to discover where they came from. On the contrary, if a clone was reverse simulated from its final spatial distribution back to the young limb bud the corresponding region on the young shape will be proportionally larger than the final clone. This is clearly the opposite of the normal forward simulation, which starts with a region much smaller than the final clone – a single triangle. This distinction is explained in more detail in Text S3. The purpose of this reverse map is therefore instead to find the full distribution of possible progenitor regions for a given final PD zone, e.g. to find the possible distribution of all zeugopod progenitors at E10. Due to the effective diffusion caused by cell mixing, the potential progenitor region for a given segment will always be proportionally larger than the final segment. As an example, if diffusion was high enough there would be a moment in the early limb bud when any single cell across the whole bud could provide descendants contributing to all 3 PD zones of the late bud. In other words, the progenitor region for any point of the older limb would be the whole young limb.

**Figure 9:**
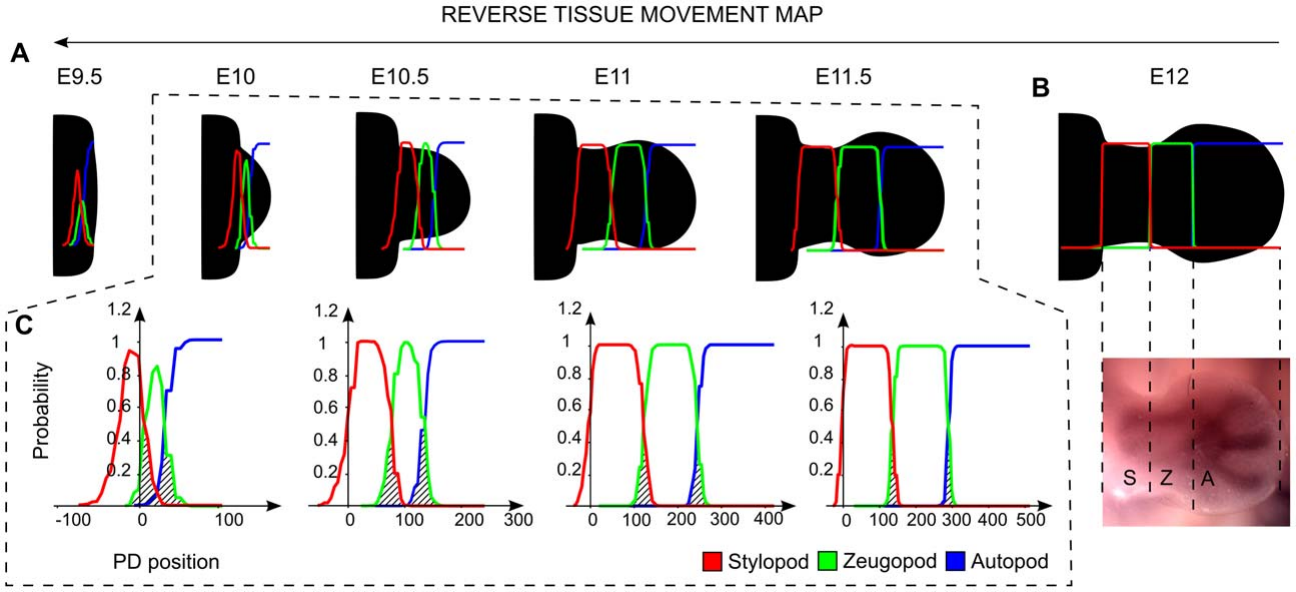
PD segments progenitors. (A) A reverse tissue movement map was calculated in order to identify the progenitor regions for the three PD segments. In the graphs, the stylopod is highlighted in red, the zeugopod in green and the autopod in blue. (B) On the top, the initial position of the three PD segments is specified as shown by an in situ hybridization at stage E12 of the Sox9 skeletal marker, on the bottom. (C) Graphs showing the retrospective probability to belong to the three segments along the proximal distal axis. The regions having a high probability to belong to more than one segment are highlighted with diagonal black lines.

The positions of the PD segments at E12 were determined by the expression of the Sox9 skeletal marker, see Figure 9B. Based on the degree of cell mixing seen in real clones, our reverse model revealed the existence of regions having high probability to contribute to two or three segments at early stages of development (around stage E10 in Figure 9C) when most of the fate maps discussed above have been performed. In other words, assuming a spatially-uniform cell mixing that matches the observed overlaps of experimental clones, our model clearly suggests that the degree of mixing between the three PD segments does not allow an early specification of the PD identities even as late as E10.

**Figure 10:**
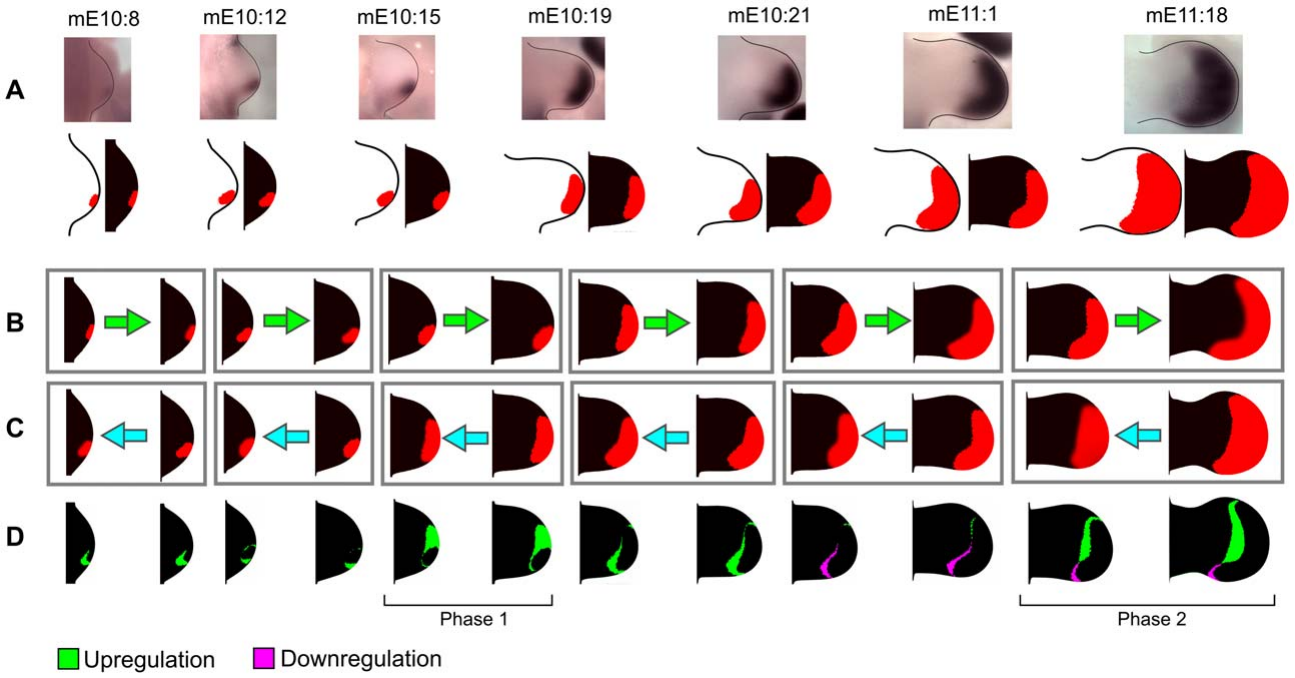
Hoxd13 domain expansion. (A) Top row: a sequence of 7 in-situ hybridizations of Hoxd13 with corresponding stage given by the morphometric staging system [17]. Bottom row: the thresholded gene expressions (limb with white background) are mapped into the triangular meshes of the model (limb with black background). (B) A set of distinct simulations (gray rectangles) showing how the gene expression domains of each time point would change if only passively carried along by tissue movements, i.e. with no active cellular up-regulation. (C) A similar set of simulations, but this time in reverse for each time point. (D) Differences between the experimental patterns and the “growth-only” simulated patterns in the correspondent column. The green regions are predicted to be up-regulated and the magenta regions to be down-regulated. This indicates that the Hoxd13 domain expansion requires active up-regulation on the anterior part around stage mE10.19 (phase 1) and on the proximal part around sage mE11∶18 (phase 2).

For the second analysis of PD patterning we investigated the possible contribution of tissue movement to the known expansion of the Hoxd13 distal marker. First we mapped into the model a gene expression time course of Hoxd13 that was obtained from in-situ hybridization at 7 different developmental stages, see Figure 10A. This was done by staging each limb with the morphometric staging system presented in [28] and by mapping the domain of expression into the correspondent time point of the model. Secondly, we used the model to expand or shrink each experimental domain of expression into the following or the previous experimental time point, see Figure 10B,C. By computing the difference between the predicted and the experimental domains we were able to disentangle the active regulation of Hoxd13 from the underlying tissue movement. Our model showed that there were periods of growth during which the change in Hoxd13 domain was fairly consistent with the underlying tissue movements. However, two particular periods stood out from this trend during which there was strong active up-regulation of the gene. The first period was from stage mE10∶15 to stage mE10∶19, when the Hoxd13 domain undergoes a quick expansion from the posterior to the anterior part of the limb, and the second period was from stage mE11∶1 to mE11∶18, when the gene was up-regulated proximally. Interestingly, these phases seemed to correlate well with (a) the up-regulation of some of the FGFs expressed by the AER that have been described to expand from the posterior to anterior part of the distal ectorderm in an initial phase around E10.5, and (b) a later phase when FGFs expression is up-regulated around stage E11∶5. These observations also fitted with the model proposed in [14] where FGF signaling was proposed as a candidate for the regulation of distal markers like Hoxa13.

In conclusion, our model predicts that the degree of mixing observed in mouse is too high to support the Early Specification Model as a realistic description of PD region specification. Moreover we have also shown that the expansion of the Hoxd13 domain, one of the genes proposed as a distal marker of the limb, cannot be explained considering tissue movement only but has to involve active up-regulation in at least two distinct phases of the development.

## Discussion

In this study we present a novel computational method which combines a sequence of experimental 2D limb morphologies and clonal data to estimate a comprehensive description of the tissue movement map responsible for limb morphogenesis. We present a mouse clonal analysis of early hind limb development and show how this allows us to estimate a 2D descriptive model of limb outgrowth that fits the experimental data. In practice, our approach is a reverse-engineering method. It is important to note that the spring analogy algorithm is used as a convenient tool for creating a variety of different hypothetical growth maps, but is not employed to represent the mechanical properties of the tissue.

A major advantage of our model over previous fate maps is the resulting comprehensive prediction of tissue movements over time and space. Previously, the behavior of a point of tissue had to be inferred by manual comparison to its closest experimental clone. By contrast, in our new map the movement of every piece of tissue is described numerically across the whole period of development. A related advantage is the temporal accuracy – the state of any hypothetical clone can be predicted at any intermediate time point – not only at the beginning or end of a virtual clonal experiment. The spatio-temporal comprehensiveness of the model gives it the power to make more concrete predictions about PD patterning. To the best of our knowledge, this is the first comprehensive 2D model of limb outgrowth derived from experimental data.

Many aspects of our clonal analysis agree with previous results in mouse [11] and with fate maps in chick [9], in particular that clones expand across one or two PD segments but never span across the whole PD axis of the limb. By measuring the degree of overlap between clones at different PD position we found that clones spanning the zeugopod had a higher degree of overlap – in fact not a single zeugopod-restricted clone was found. A quantification of the ratio between AP and PD clone lengths highlighted a range of behaviors, but which could be broadly split into two type: isotropic clones on the distal and proximal part of the limb, and anisotropic clones in the bulk of the tissue showing greater PD length than AP length (Figure 3). Interestingly, in contrast to fate maps in chick [8], [9], we found that some distal clones expanded more on the AP axis than the PD axis (e.g. C2 in Figure 5A). This was also reflected in the fitting of the hypothetic growth maps to the clonal data. The map which fitted best (Map6) displayed specific features regarding AP and PD growth: along the PD axis it was one of the maps with a distally-restricted PD elongation. On its own, this information would appear to contradict the knowledge that proliferation rates are highest distally, however Map6 was also the one with a strong distal “fanning-out” movement along the AP axis (central row in Figure 4B). This compensates the low PD expansion resulting in a strong AP-oriented anisotropy, such that predicted proliferation rates are maintained at high levels in this region (see Figure 8). Interestingly, although this feature may be stronger in mouse than chick (resulting in a wider mouse autopod) recent reports have suggested that the AP expansion of the chick and gecko autopod could be driven at least in part by AP-oriented cell divisions [33]. Taking advantage of our computer model we can calculate the growth tensors directly revealing the AP anisotropy of the distal tissue. However, the tensors also show that tissue movements of the most anterior and the most posterior regions of the sub-ridge mesenchyme are perpendicular to the AER, rather than parallel (Figure 8C). This is an unexpected observation that will require more attention in future studies.

Another interesting observation regards the general construction of our model. By representing the local density of labeled cells as a probability distribution which can diffuse through a smoothly deforming mesh, we shows that biologically-realistic tissue movements can be captured through the combination of anisotropic velocity vector field, with isotropic diffusion. This could suggest that the cellular properties which govern mixing, such as cell-cell adhesion, may not themselves display any cell polarity. In other words, it is theoretically plausible that cells are subject to two types of activity: directional movements (such as oriented cell divisions or convergent extension) which are responsible for the tissue-level shape changes, and non-directional cell mixing. However, in reality, alternative scenarios may also be equally compatible with our model. For example, it is likely that oriented movements naturally lead to the intercalation and therefore to the mixing of cells, such that directional movement and cell mixing cannot be conceptually uncoupled.

Finally, we used the model to clarify the relation between mouse limb tissue movement and the existing PD patterning hypothesis. Firstly we showed, by using a reverse version of the model, that there is a considerable degree of mixing between the progenitors of the three PD segments (Figure 9). In contrast to the Early Specification Model, our model predicts that at early stages there are regions where cells have a high probability to contribute to more than one PD segment. Secondly we showed, by mapping a time course of Hoxd13 expression in the model, that the expansion of the Hoxd13 domain cannot be explained only by tissue movement but requires active gene regulation, Figure 10. The model gave specific predictions of the type of spatial and temporal active regulation of Hoxa13 required suggesting, as proposed in [14], that distal markers could be under the control of the FGF signaling coming from the AER. Taken together these two results prove that limb growth modeling is a valuable resource to extract maximum information from clonal data and to make specific predictions about the spatio-temporal dynamic of limb morphogenesis.

To conclude, the software that we developed will allow us to easily integrate, inside a realistic 2D model of limb growth, numerical simulations of gene regulatory networks and morphogen gradients taking a big step forward in the study of limb development by using a systems biology approach.

## Materials and Methods

### Ethics statement

All animals were handled in strict accordance with good animal practice as defined by the relevant national and/or local animal welfare bodies, and all animal work was approved by the appropriate committee.

### Clonal data

The clonal data was produced using the tamoxifen inducible Cre-line presented in [11]. Lineage tracing at clonal resolution was obtained by injecting low dose of tamoxifen (0.10mg) to reduce the probability of polyclonal origin as described in [11]. Female pregnant females were injected at approximately 8 days pf. and embryos were extracted at 12 pf. LacZ clone staining was performed as described in [34] and embryos were post-fixed in 4% para-formaldehyde (PFA) and stored in 80% glycerol, 4% PFA.

### Limb morphologies

72 limb morphologies were extracted from an extended version of the standard morphological trajectory presented in [28]. Each limb morphology was represented by an array of curvature values that were averaged from more than 600 outlines of real limb buds at different stages. We geometrically reconstructed 72 limb shapes from the standard morphological trajectory and added an artificial body of 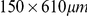 to each shape, see Figure S1. We thus spatially discretized each of the limb bud shapes using the unstructured triangular mesh generator presented in [35]. The meshes used to build the 9 tissue movement maps in Figure 4 were obtained with an element length of 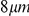 and the finer version of the meshes in Figure S2 with an element length of 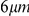.

### Gene expression data

The Hoxd13 gene expression time course in Figure 10 and the Sox9 expression in Figure 3 were made using C57Bl/6J mouse embryos, fixed in 4%PFA and dehydrated into MetOH. Whole Mount In situ hybridizations were stained using NBT/BCIP and performed using a Hoxd13 antisense probe, kindly provided by Denis Duboule, and a Sox9 antisense probe.

### Virtual tissue movment maps

Software to generate the virtual tissue movement maps was written in Java and used the free visualization library vtk [36] to implement the graphical user interface that allowed the user to specify the *boundary control splines*. Virtual tissue movement maps were stored on a re-usable data structure called *MorphoMovie* that was defined by a series of velocity vector fields and a series of triangle interpolation maps, one for each triangular mesh in the sequence. We developed a generic partial differential equation solver that was able to simulate virtual clones on a given *MorphoMovie*. The solver used an Euler method for time discretization and a Finite Volume Method [37], [38] on unstructured triangular meshes for the space discretization.

## Supporting Information

Figure S1 Standard morphological trajectory. The 72 experimental limb bud morphologies describing mouse hind-limb development from stage E9 to stage E12.
(0.33 MB PDF)

Figure S2 Analysis of the clonal data (PD and AP length measurement). The clonal data that was generated using the tamoxifen inducible CRE transgenic mouse line. Clones are divided in two groups: isotropically expanding clones and an-isotropically expanding clones. The PD and AP clone lengths relative to the maximum PD and AP length of the limb are measured. Colored triangles represent the AP and PD clone expansion.
(1.17 MB PDF)

Figure S3 Proliferation patterns of the maps shown in Figure 4. The proliferation patterns of the 9 virtual tissue movement maps in Figure 4. Triangle expansion rate are converted to cell cycle time and are visualized by using a heat map, red corresponds to low cell cycle times (11h) and blue to high cell cycle times (43h).
(0.40 MB PDF)

Figure S4 Experimental clone registration. The collection of 13 experimental clones that were mapped into the last triangular mesh of the sequence (stage E12).
(0.66 MB PDF)

Figure S5 Clone scores of the tissue movement maps in Figure 4. For each of the 9 virtual tissue movement maps, the collection of virtual clones that best matched the 13 experimental clones. Virtual clones are shown with colored contour lines and experimental clones are shown in white color. The text in white color is the virtual clone score. Finally, a bar diagram summarizing the clone scores.
(1.68 MB PDF)

Text S1 Description of the tissue movement maps in Figure 4. A description of the spline curves and stiffness distributions used to generate the 9 virtual tissue movement maps in Figure 4.
(0.04 MB PDF)

Text S2 Simulation with a refined triangular mesh. The 13 virtual clone positions in Map6 that best matched the experimental data were used to simulate virtual clones on a version of Map6 with a refined mesh.
(0.46 MB PDF)

Text S3 Forward and backward maps. A description of the different information that can be extrapolated from a virtual tissue movement map: fate maps vs progenitor regions. The PD segment progenitor prediction shown in Figure 9 is also recalculated by using Map6 without additional diffusion.
(1.00 MB PDF)

Video S1 Mesh deformation video. Part of the sequence of triangular meshes that was derived from the experimental limb bud morphologies. Each mesh is deformed to match the next mesh in the sequence from which the simulation continues.
(5.46 MB AVI)

Video S2 Virtual fate map video. A video showing a virtual fate map. The spatial probability distribution of the fate is colored with a heat map, red represents a high probability and blue a low probability. A discrete number of triangle-interpolation steps can be appreciated in the video.
(3.21 MB AVI)

Video S3 Video of the simulation in Figure 7B. A video showing a number of clones that match the distribution and shape of the experimental clonal data. Blue clones expand isotropically on the AP and the PD axis, red and green clones expand more on the PD axis.
(2.70 MB AVI)
