# Supplementary figures and images for: A Computational Clonal Analysis of the Developing Mouse Limb Bud
Source: PLoS Comput Biol. 2011 Feb 10;7(2):e1001071. doi: 10.1371/journal.pcbi.1001071 (PMC3037386; doi:10.1371/journal.pcbi.1001071)

**Figure S1: The standard morphological trajectory – from E9 to E12**

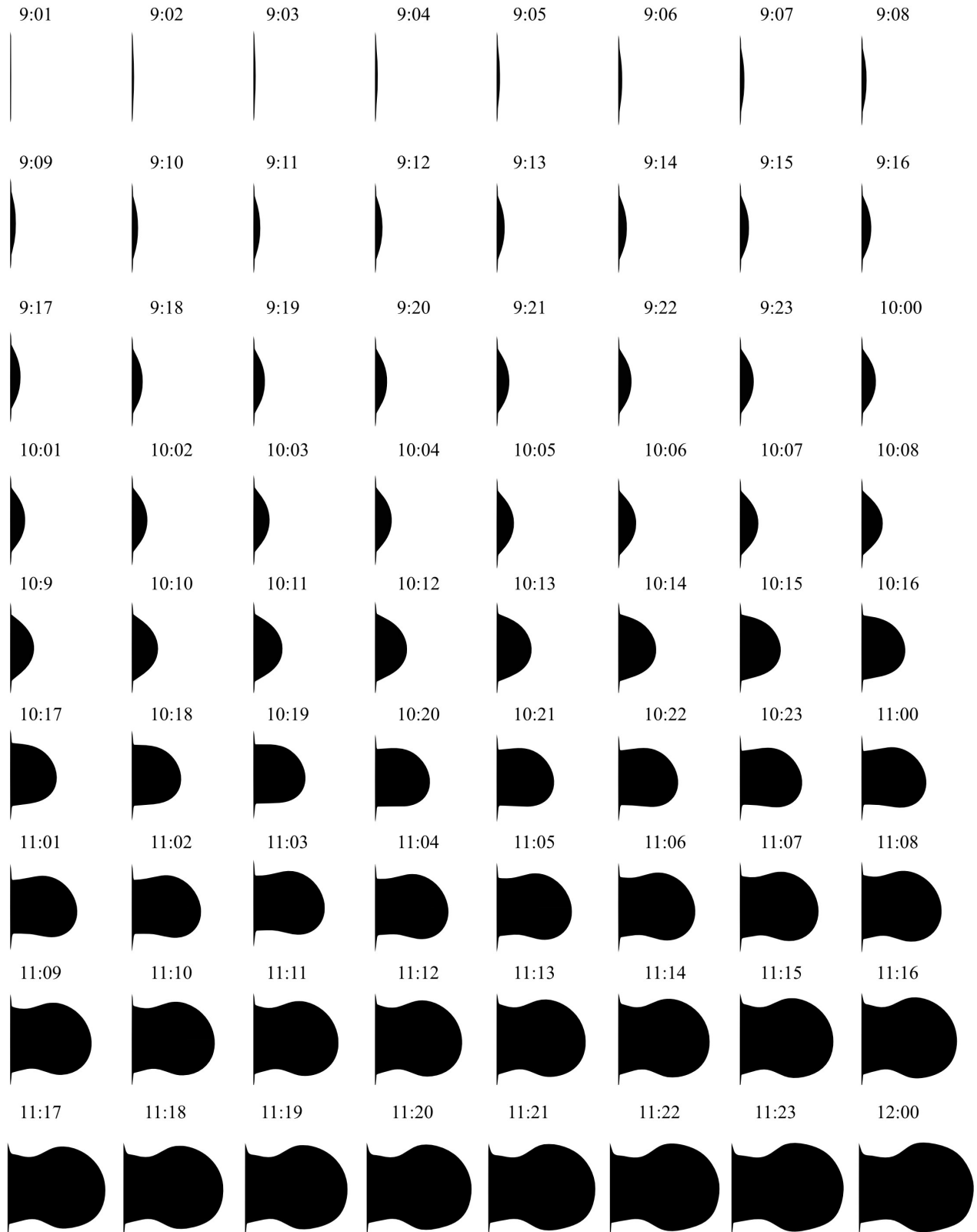

Supplement: Figure S1 — Standard morphological trajectory. The 72 experimental limb bud morphologies describing mouse hind-limb development from stage E9 to stage E12. (0.33 MB PDF) [file pcbi.1001071.s001.pdf]

**Figure S4: Experimental clone registration, triangular mesh at E12**

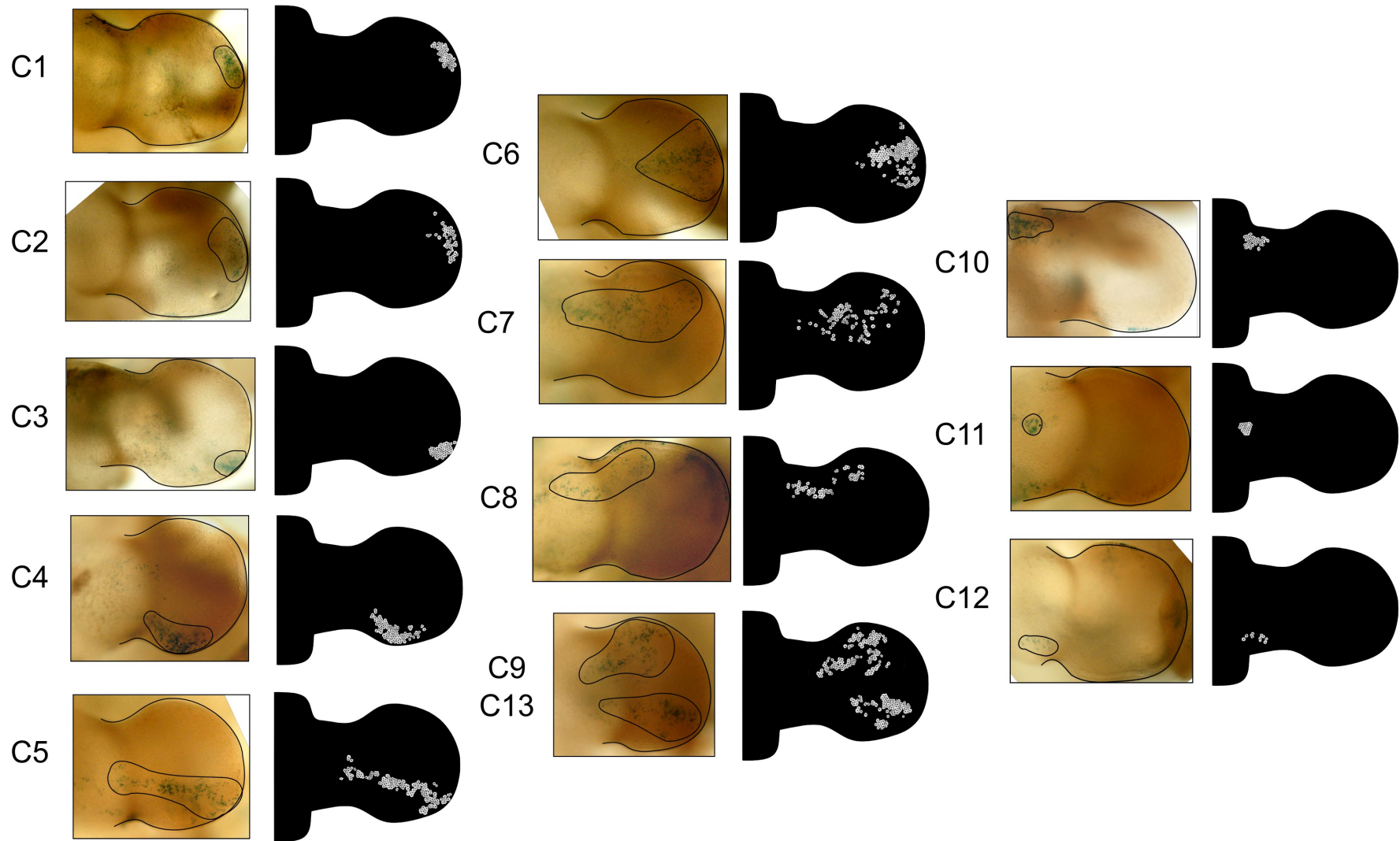

Supplement: Figure S4 — Experimental clone registration. The collection of 13 experimental clones that were mapped into the last triangular mesh of the sequence (stage E12). (0.66 MB PDF) [file pcbi.1001071.s004.pdf]

**Figure S5: Clone scores of the tissue movement maps in Figure 4**

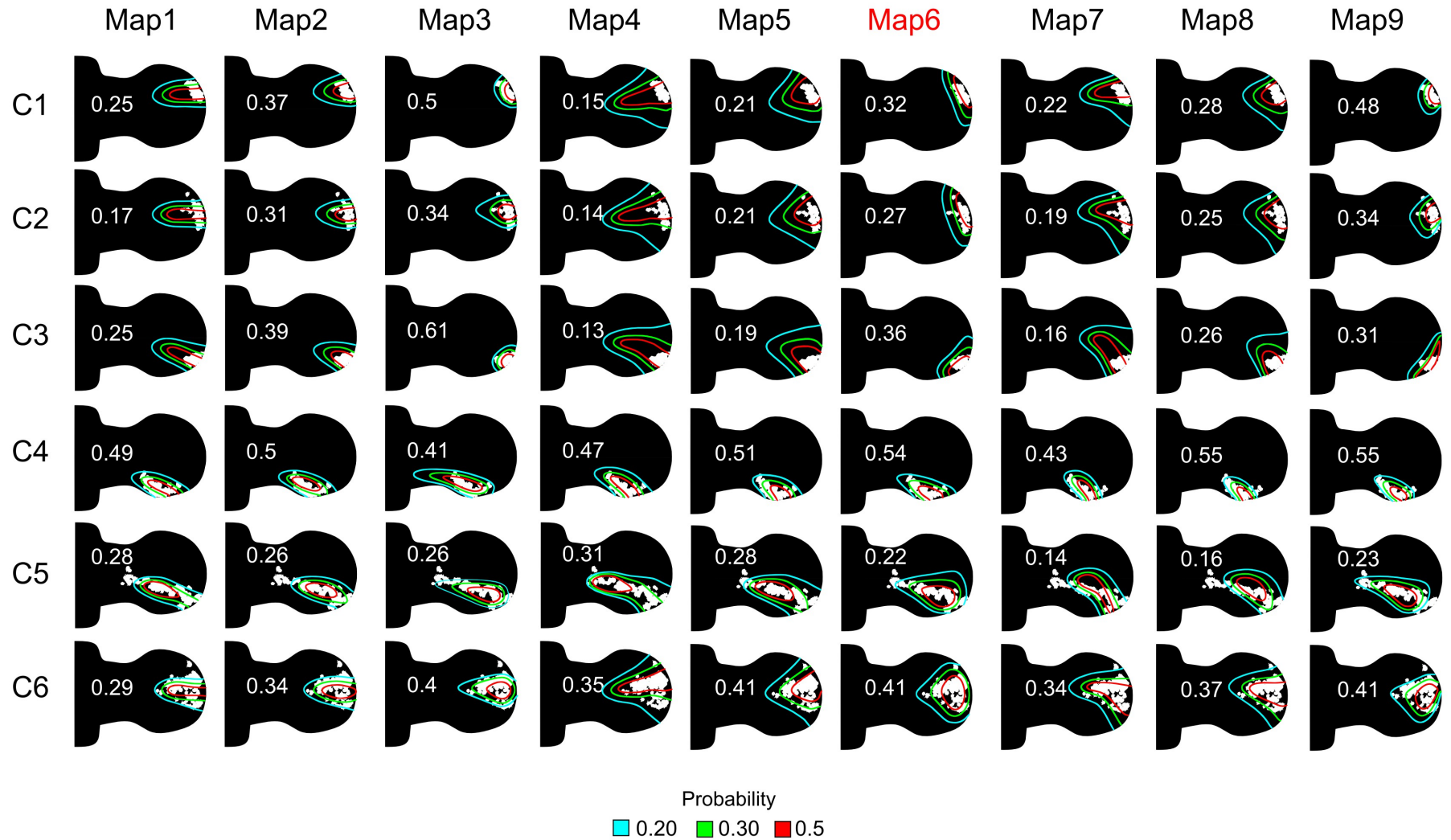

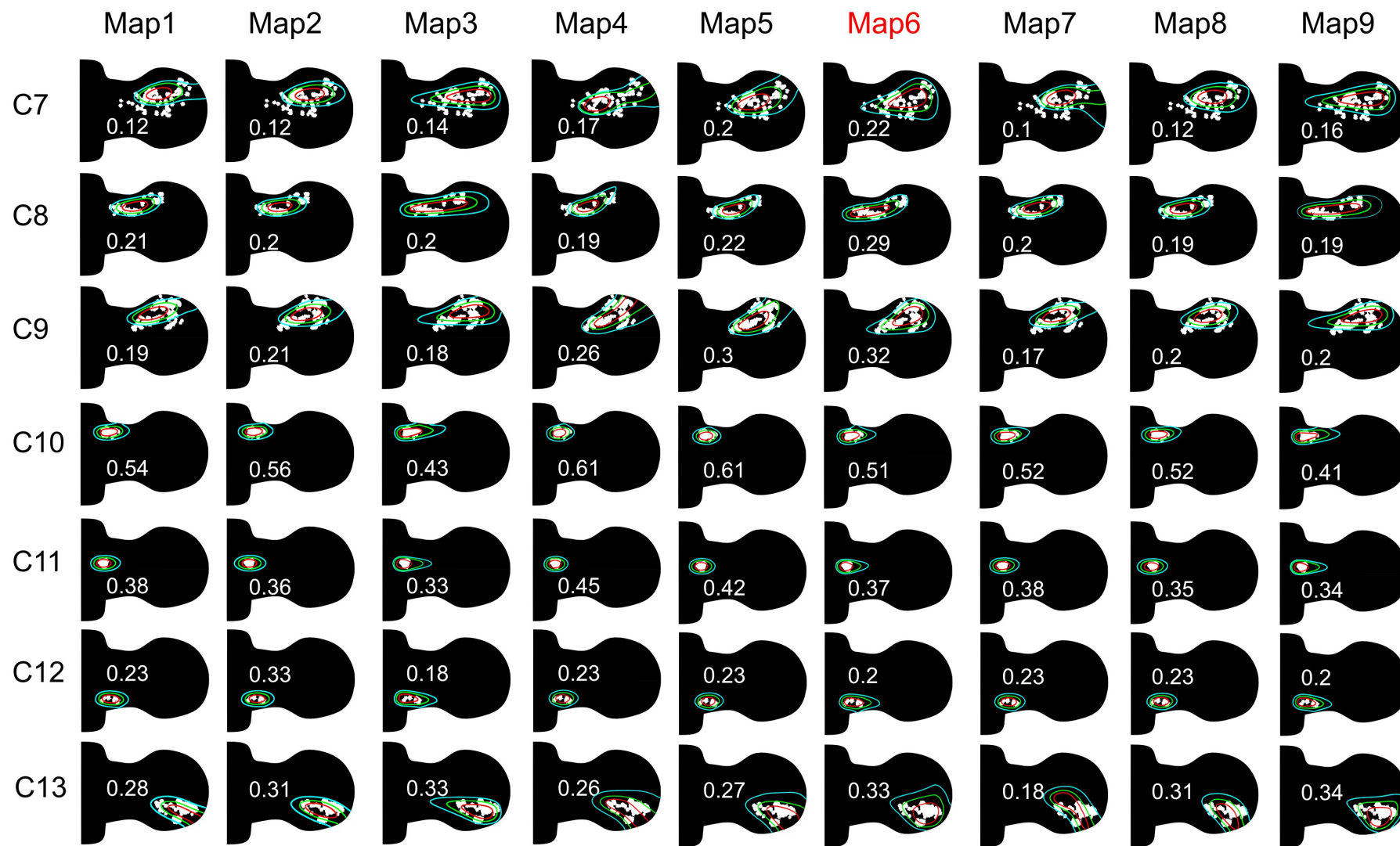

Probability  
■ 0.20 ■ 0.30 ■ 0.5

## Summary

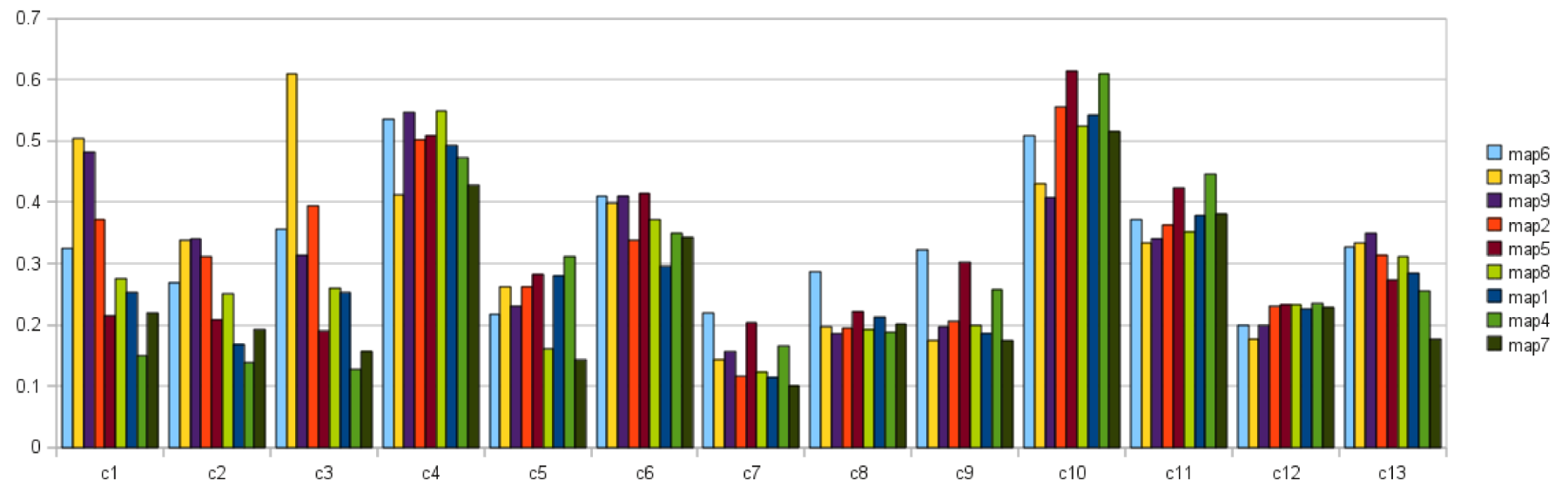

Supplement: Figure S5 — Clone scores of the tissue movement maps in Figure 4. For each of the 9 virtual tissue movement maps, the collection of virtual clones that best matched the 13 experimental clones. Virtual clones are shown with colored contour lines and experimental clones are shown in white color. The text in white color is the virtual clone score. Finally, a bar diagram summarizing the clone scores. (1.68 MB PDF) [file pcbi.1001071.s005.pdf]
