# Supplementary material for: A Computational Clonal Analysis of the Developing Mouse Limb Bud
Source: PLoS Comput Biol. 2011 Feb 10;7(2):e1001071. doi: 10.1371/journal.pcbi.1001071 (PMC3037386; doi:10.1371/journal.pcbi.1001071)
Supplement: Figure S2 — Analysis of the clonal data (PD and AP length measurement). The clonal data that was generated using the tamoxifen inducible CRE transgenic mouse line. Clones are divided in two groups: isotropically expanding clones and an-isotropically expanding clones. The PD and AP clone lengths relative to the maximum PD and AP length of the limb are measured. Colored triangles represent the AP and PD clone expansion. (1.17 MB PDF) [file pcbi.1001071.s002.pdf]

A computational clonal analysis of the developing mouse limb bud
Marcon L. et al.

**Figure S2: Analysis of the clonal data (PD and AP length measurement)**

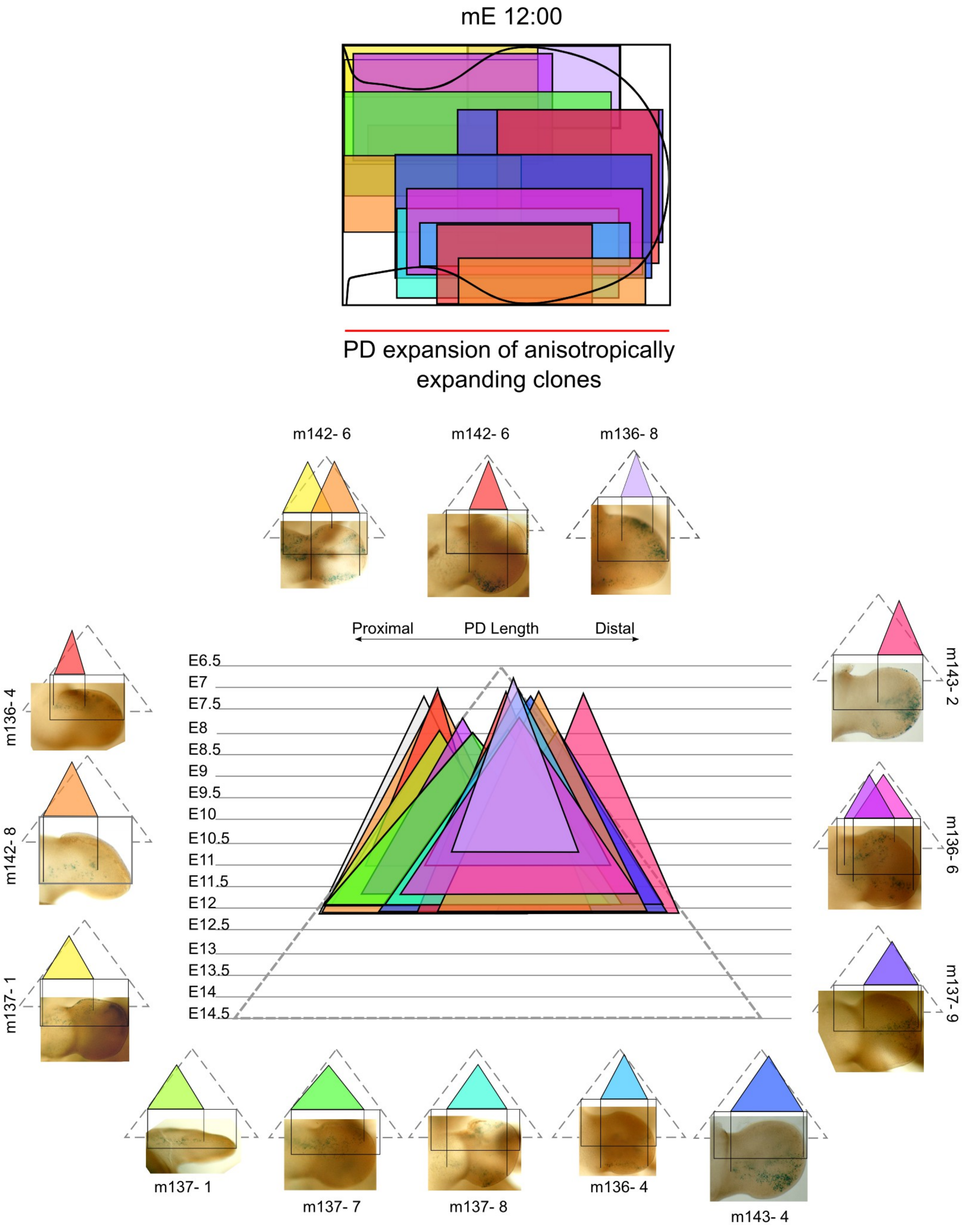

mE 12:00

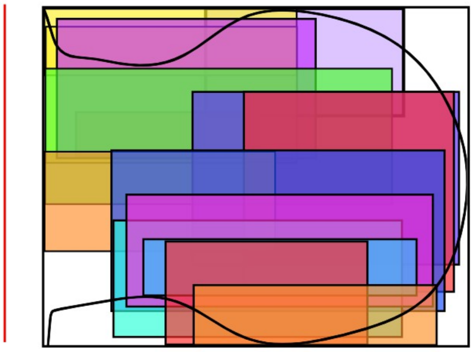

AP expansion of anisotropically expanding clones

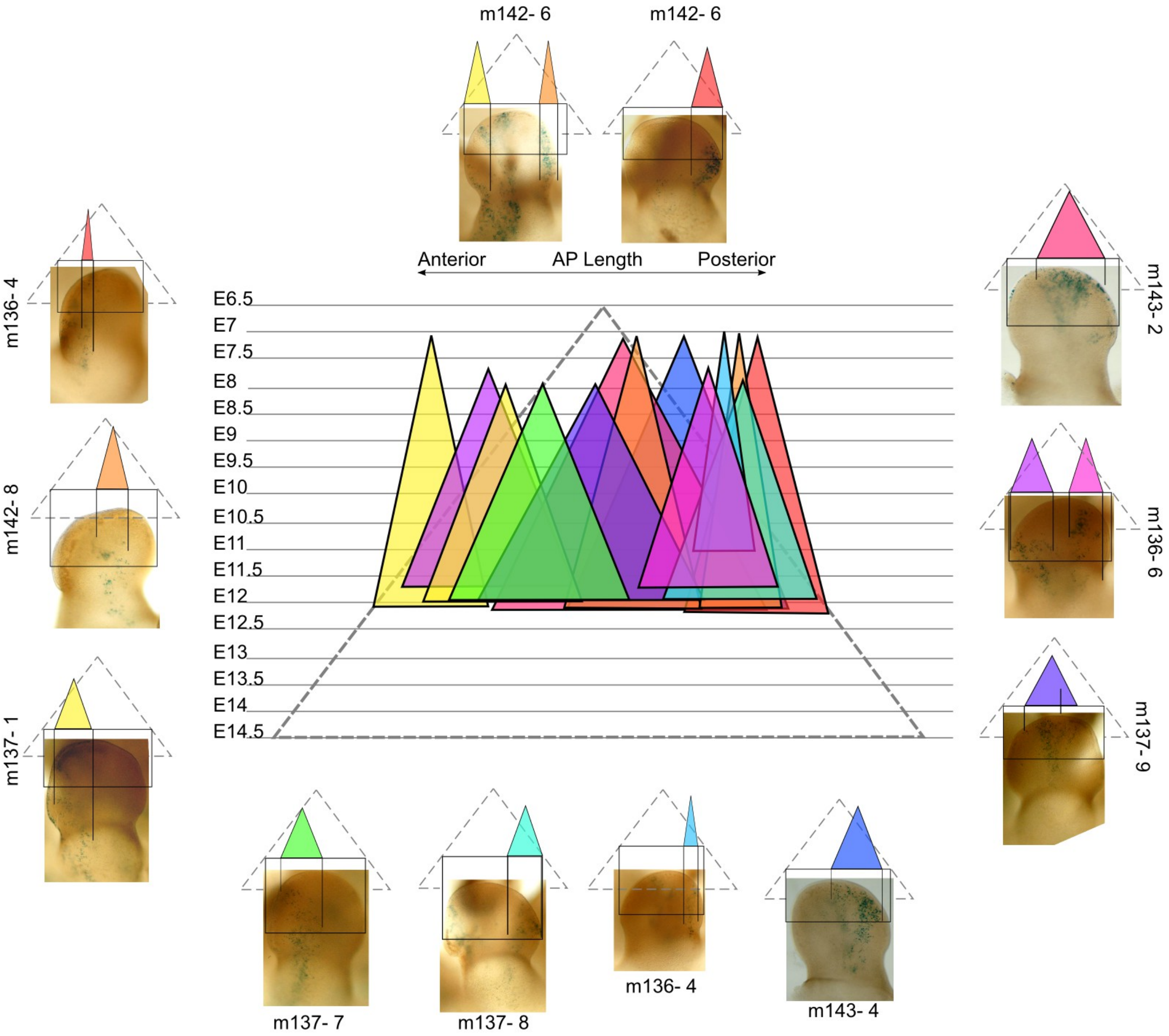

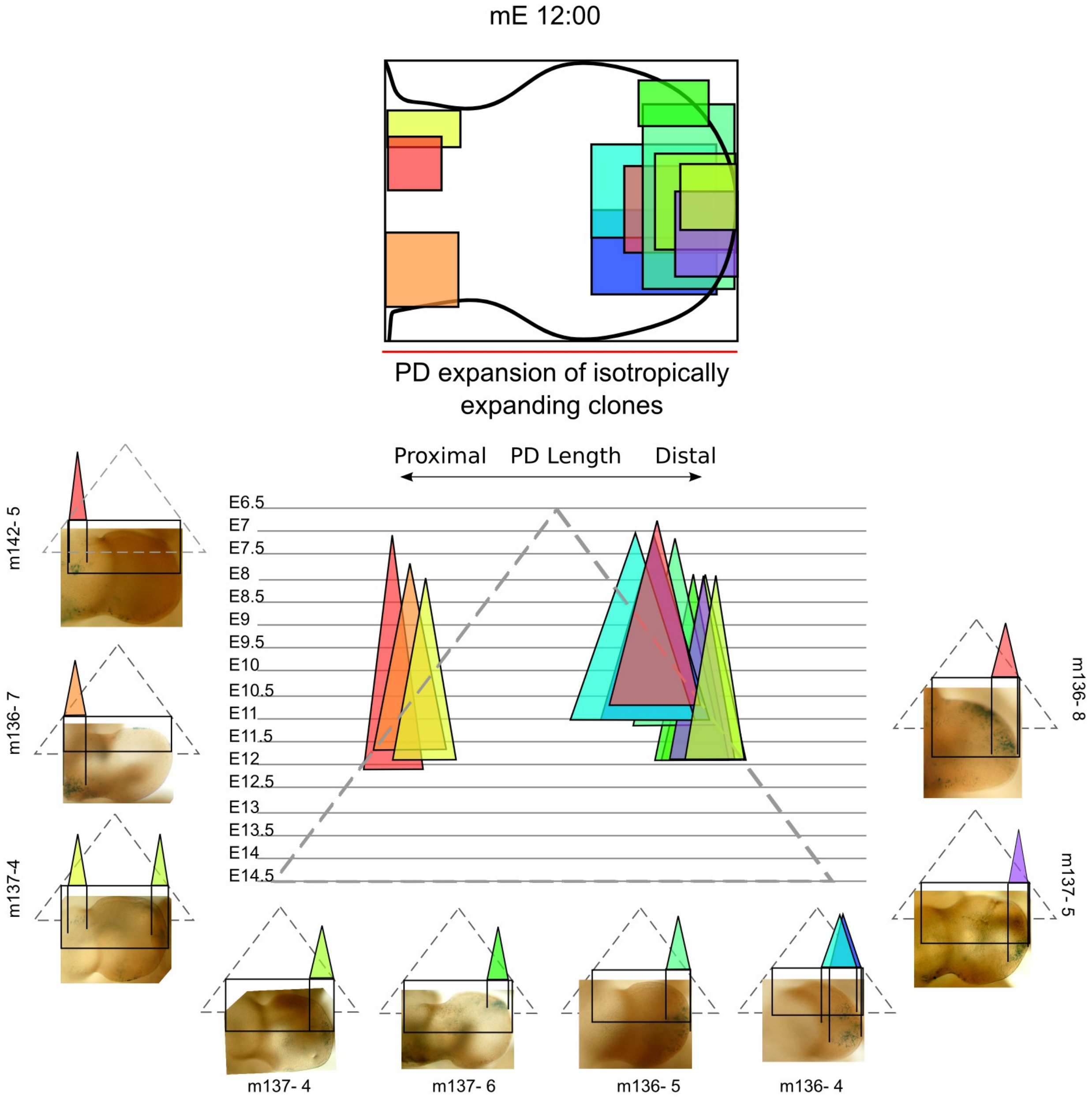
mE 12:00
PD expansion of isotropically expanding clones
Proximal
PD Length
Distal
E6.5
E7
E7.5
E8
E8.5
E9
E9.5
E10
E10.5
E11
E11.5
E12
E12.5
E13
E13.5
E14
E14.5
m142- 5
m136- 7
m137-4
m136- 8
m137- 5
m137- 4
m137- 6
m136- 5
m136- 4

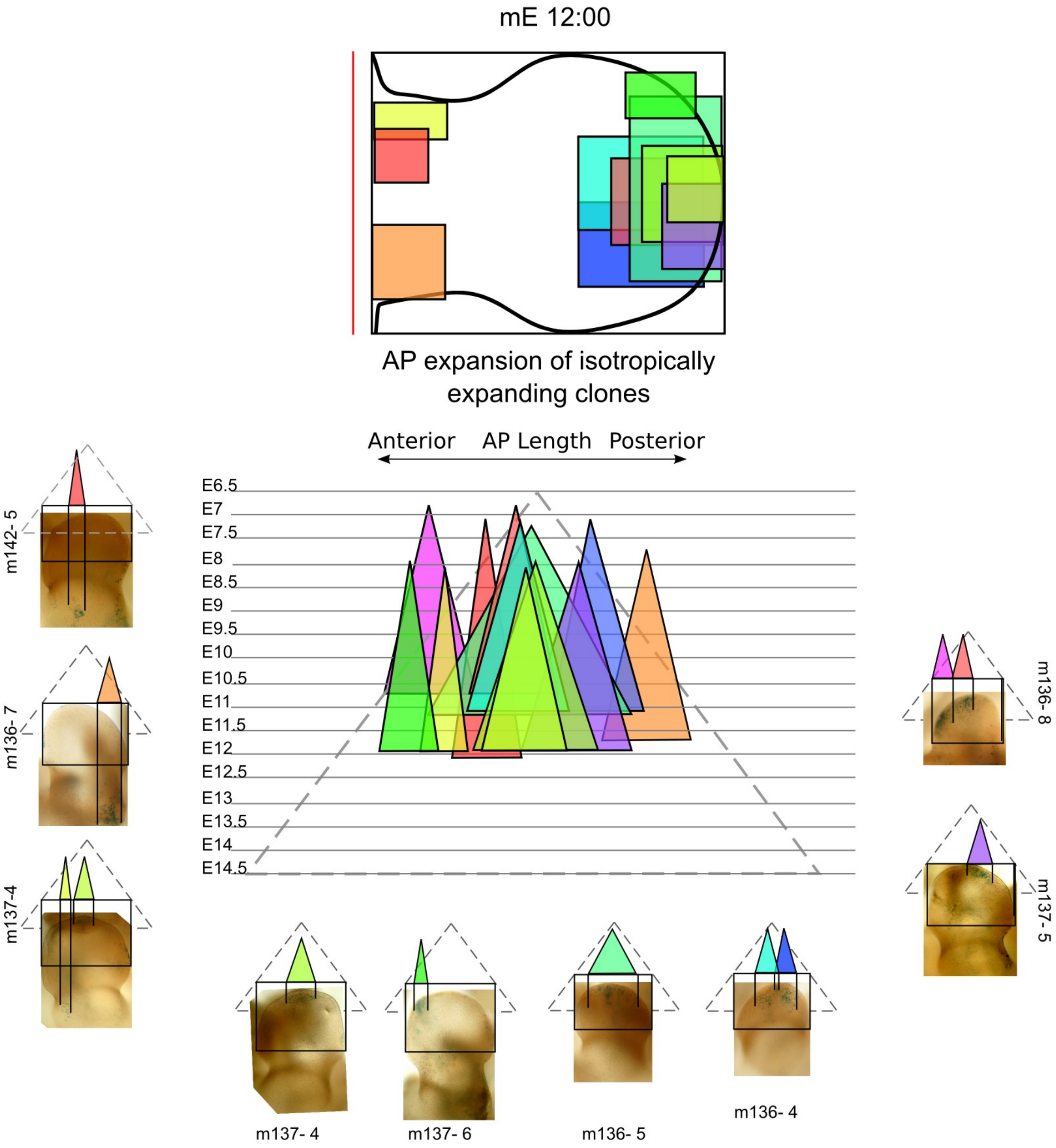

mE 12:00
AP expansion of isotropically expanding clones
Anterior
AP Length
Posterior
E6.5
E7
E7.5
E8
E8.5
E9
E9.5
E10
E10.5
E11
E11.5
E12
E12.5
E13
E13.5
E14
E14.5
m142- 5
m136- 7
m137-4
m136- 8
m137- 5
m137- 4
m137- 6
m136- 5
m136- 4
