# Supplementary material for: A Computational Clonal Analysis of the Developing Mouse Limb Bud
Source: PLoS Comput Biol. 2011 Feb 10;7(2):e1001071. doi: 10.1371/journal.pcbi.1001071 (PMC3037386; doi:10.1371/journal.pcbi.1001071)

A computational clonal analysis of the developing mouse limb bud
Marcon L. et al.

Figure S3: Proliferation patterns of the maps shown in Figure 4

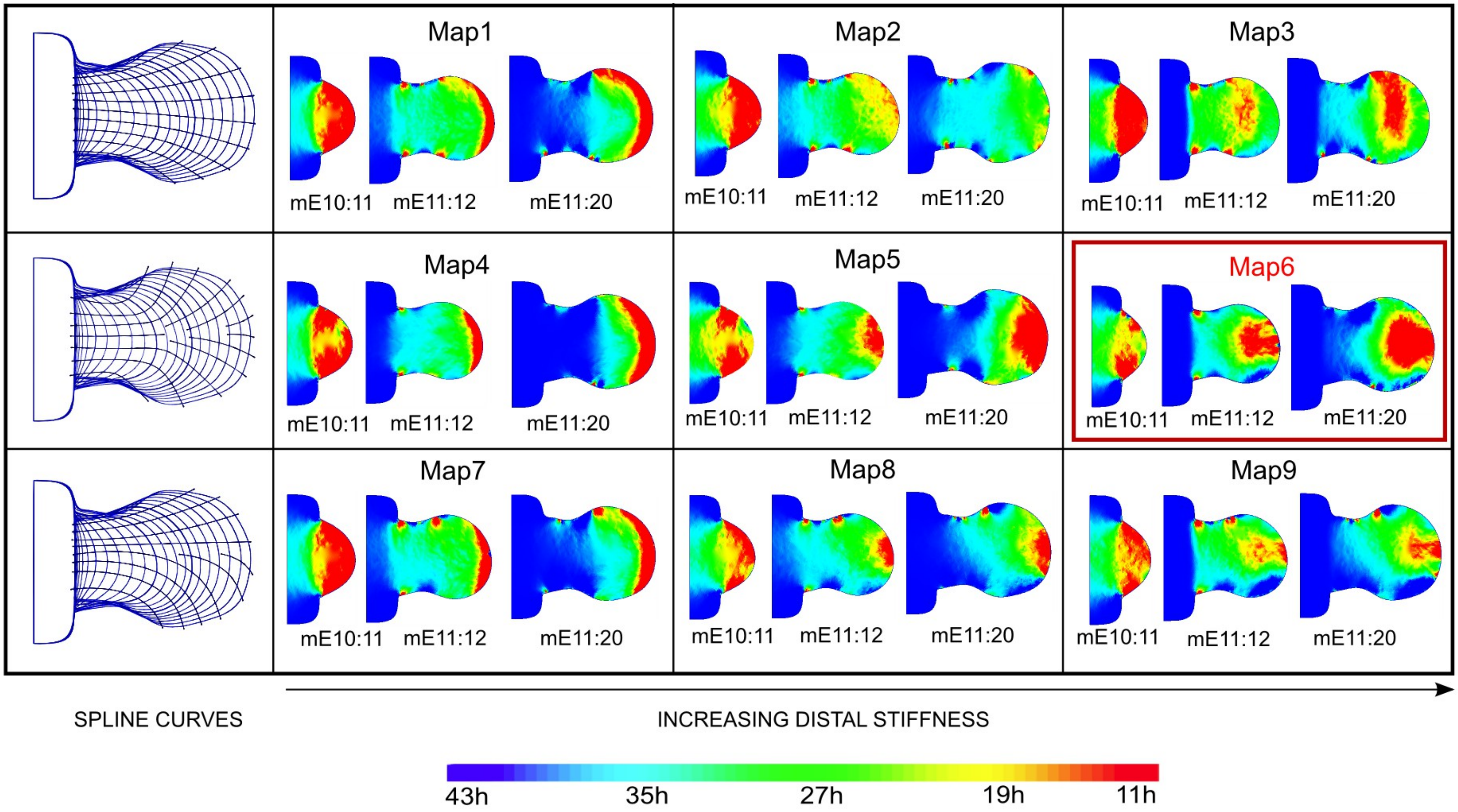

Supplement: Figure S3 — Proliferation patterns of the maps shown in Figure 4. The proliferation patterns of the 9 virtual tissue movement maps in Figure 4. Triangle expansion rate are converted to cell cycle time and are visualized by using a heat map, red corresponds to low cell cycle times (11h) and blue to high cell cycle times (43h). (0.40 MB PDF) [file pcbi.1001071.s003.pdf]
