# Supplementary material for: A Computational Clonal Analysis of the Developing Mouse Limb Bud
Source: PLoS Comput Biol. 2011 Feb 10;7(2):e1001071. doi: 10.1371/journal.pcbi.1001071 (PMC3037386; doi:10.1371/journal.pcbi.1001071)
Supplement: Text S1 — Description of the tissue movement maps in Figure 4. A description of the spline curves and stiffness distributions used to generate the 9 virtual tissue movement maps in Figure 4. (0.04 MB PDF) [file pcbi.1001071.s006.pdf]

A computational clonal analysis of the developing mouse limb bud
Marcon L. et al.
Text S1: Description of the tissue movement maps in Figure 4

Tissue movement maps were generated by varying the directionality and the relative magnitude of the velocity vectors. A total of 9 maps (shown in Figure 4) were generated by using three different sets of spline curves and three different distributions of stiffness coefficients along the proximal-distal axis.

The three maps in the first row of Figure 4 (Map1, Map2, Map3) were obtained by using a set of spline curves that defined velocity vector fields with direction bias to the distal tip of the limb. The three maps of the second row (Map4, Map5, Map6) were obtained using a set of spline curves that defined velocity vectors that were progressively spreading-out more along the anterior-posterior axis of the limb. Finally, the spline curves that were used to generate the maps of the third row (Map7, Map8, Map9) defined a more asymmetric velocity vector field with posterior vectors bending to the posterior part of the limb and anterior vectors biased distally.

For each triangular mesh in the chronological sequence, edge stiffness coefficients were calculated using the formula:

$$\alpha_{ij} = \frac{1}{l_{ij}} P(e_x^{ij}) \tag{1}$$

where l_{ij} is the length of the edge connecting the vertex i and the vertex j and $P(e_x^{ij})$ is a scaling function P calculated on the x coordinate of the edge midpoint e_x^{ij}.

The three maps in the first column of Figure 4 (Map1,Map4,Map7) were generated using an inverted sigmoid as the scaling function P:

$$P(x) = \frac{1}{(1 + e^{-K_s * (-x + (m_x - d_x))})} \tag{2}$$

where K_s is a constant defining the steepness of the sigmoid, m_x is the maximum x coordinate of the mesh and d_x is a parameter used to translate the sigmoid proximally. This scaling function defined lower stiffness values for edges that were located distally, therefore allowing greater deformations on the distal part of the limb mesh.

The three maps in the second column of Figure 4 (Map2,Map5,Map7) were generated considering no change in stiffness coefficients along the proximal-distal

axis ($P(x) = 1$).

Finally, the three maps in the third column of Figure 4 (Map3,Map6,Map9) were generated using a sigmoid function P that was defined similarly to the scaling function (2) as:

$$P(x) = 1 + \frac{1}{(1 + e^{-K_s*(x-(m_x-d_x))})} \tag{3}$$

This scaling function defined higher stiffness coefficients for edges located distally, therefore allowing greater deformations on the proximal part of the mesh.

We also performed simulations using exponential and linear scaling function with similar monotonic behaviors to equations (2) and (4) and found similar mesh deformations in the first case but almost no change in mesh deformation from the constant fucntion ($P(x) = 1$) in the second case. This suggested that a non-linear scaling of the stiffness was essential to obtain a substantial difference in the mesh deformation.

The simulations that are shown in Figure 4 were performed using $K_s = 0.05$ and $d_x = 30$ and similar values of K_s and d_x showed little effect on the type of final deformation that was obtained. In addition in all the cases we also specified a greater stiffness for the part of the meshes correspondendt to the body. The space unit was μm and the maximum PD length given by the last mesh in the sequence was of $786 \mu m$.
