# Supplementary material for: A Computational Clonal Analysis of the Developing Mouse Limb Bud
Source: PLoS Comput Biol. 2011 Feb 10;7(2):e1001071. doi: 10.1371/journal.pcbi.1001071 (PMC3037386; doi:10.1371/journal.pcbi.1001071)
Supplement: Text S2 — Simulation with a refined triangular mesh. The 13 virtual clone positions in Map6 that best matched the experimental data were used to simulate virtual clones on a version of Map6 with a refined mesh. (0.46 MB PDF) [file pcbi.1001071.s007.pdf]

A computational clonal analysis of the developing mouse limb bud
Marcon L. et al.

Text S2: Simulation with a refined triangular mesh

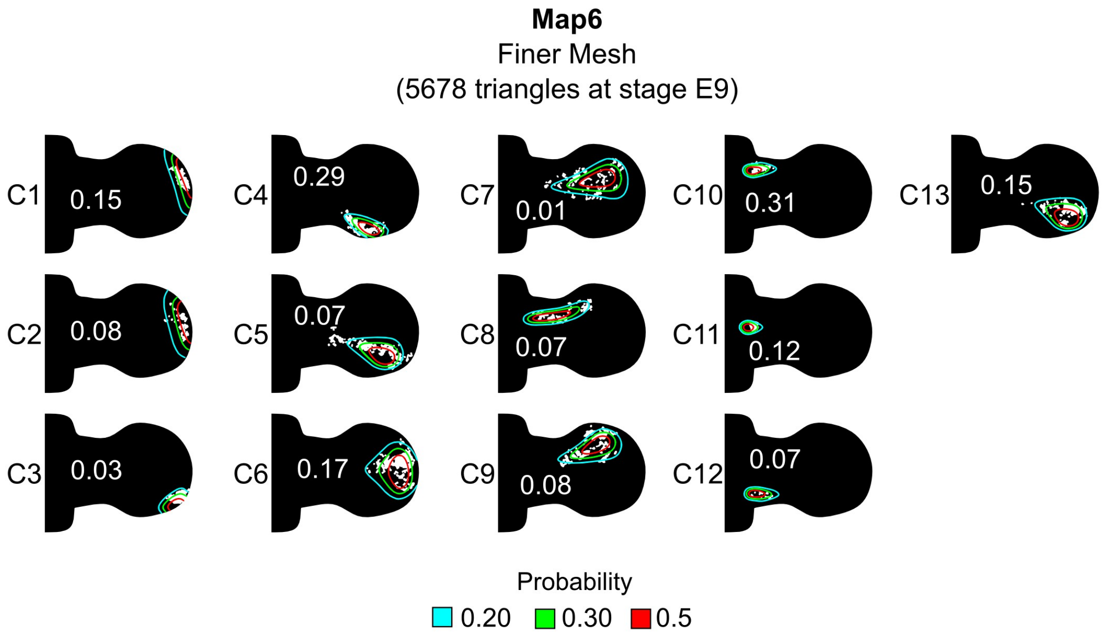

A version of Map6 with a finer mesh was generated. Using the initial positions that were optimized for the version of Map6 obtained with a coarser mesh, we computed and evaluated the 13 virtual clones on the finer mesh. Results were in agreement with the virtual clones obtained with the original mesh.

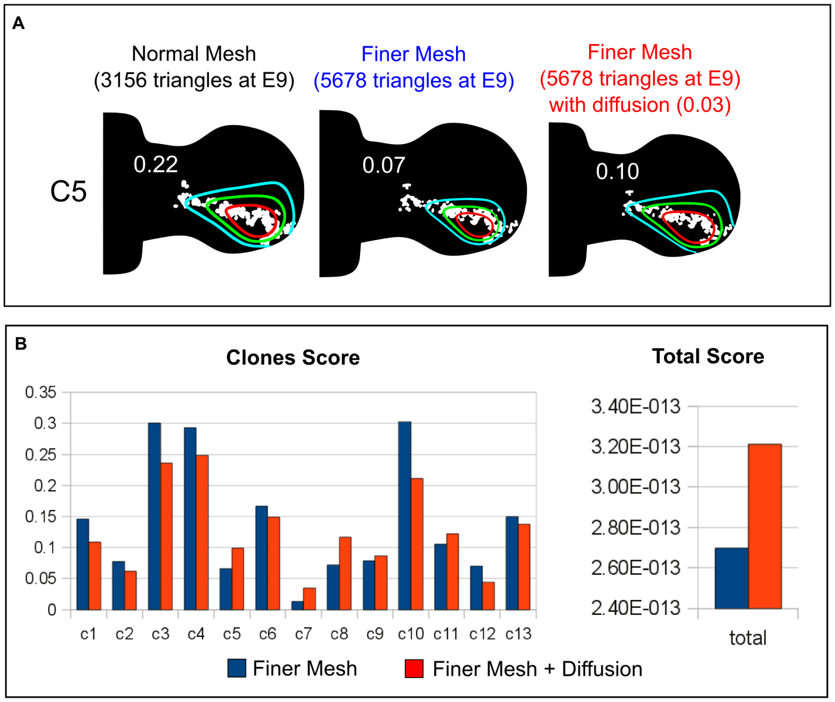

A) Example of clone generated with the Map6 on a finer Mesh and with Map6 on a finer mesh plus an additional diffusion term increasing cell mixing. **B)** Clone scores and Total score for Map6 on the finer mesh with and without diffusion.
