# Supplementary material for: A Computational Clonal Analysis of the Developing Mouse Limb Bud
Source: PLoS Comput Biol. 2011 Feb 10;7(2):e1001071. doi: 10.1371/journal.pcbi.1001071 (PMC3037386; doi:10.1371/journal.pcbi.1001071)
Supplement: Text S3 — Forward and backward maps. A description of the different information that can be extrapolated from a virtual tissue movement map: fate maps vs progenitor regions. The PD segment progenitor prediction shown in Figure 9 is also recalculated by using Map6 without additional diffusion. (1.00 MB PDF) [file pcbi.1001071.s008.pdf]

A computational clonal analysis of the developing mouse limb bud

Marcon L. et al - **Text S3: Backward and Forward Maps**

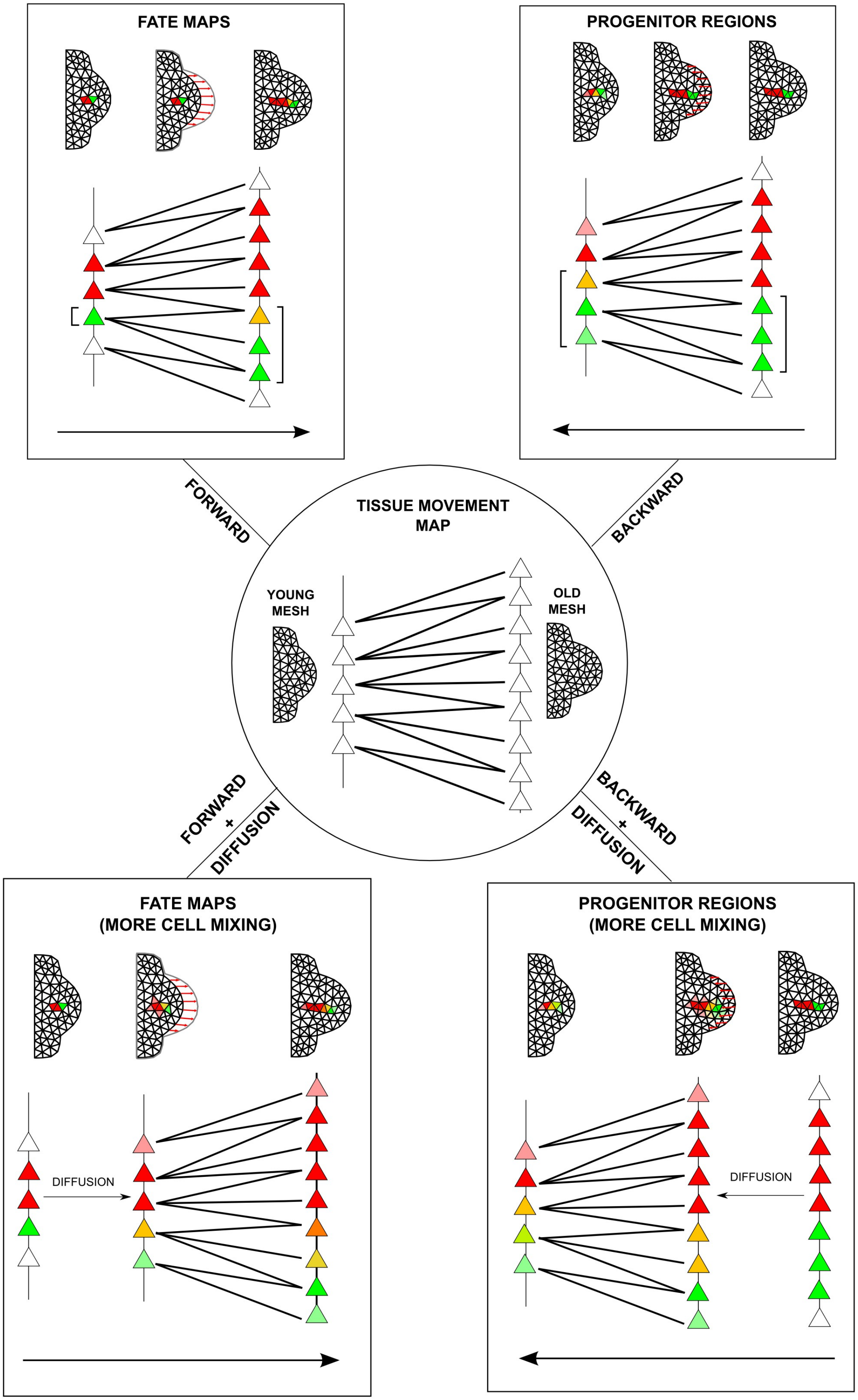

The above figure illustrates the type of information that can be obtained from a virtual tissue movement map. Inside the central circle, a tissue movement map is represented by a simplified mapping of 5 young-mesh triangles to 9 old-mesh triangles, based on their geometric overlap. This triangle map was generated starting from a velocity vector field of a hypothetical growth transform and provides information about the fate of each part of tissue (triangles) in the period of development between the two morphologies.

A tissue movement map of this type can be read in two different ways:

By reading it forward (*upper left-hand panel*) virtual fate maps can be performed by marking young-mesh triangles with a virtual dye and then observing the dye redistribution into the old-mesh triangles. In other words we use the map to explore the question: where can tissue in a triangle of the young mesh end up in the old mesh? In the specific example 2 triangles have been mapped with a red dye and 1 with a green dye. The map predicts that the red region will expand to occupy 5 triangles of the old mesh and the green region will grow into 3 triangles on the old mesh, one of which is in common between the two (yellow triangle). In biological terms the yellow triangle will contain cells labeled with green and red dye.

Conversely by reading it backwards (*upper right-hand panel*) progenitor regions can be identified by marking with a virtual dye a tissue region on the old mesh and then using the reversed triangle map to reveal the triangles from which this part of tissue could descend. In other words we are exploring the question: from which young triangles can an old triangle possibly descend? In the specific example, four triangles have been marked in the old mesh with a red dye and three triangles with a green dye. Redistributing the dye by using the reversed triangle map three triangles are predicted as the possible progenitors of the green marked tissue and three triangles as the possible progenitors of the red marked tissue, one of the triangles (in yellow) could be a progenitor of both regions.

These two interpretations are based on same triangle map but address two profoundly distinct questions. It is important to highlight that the reverse map does not provide a way to reverse a fate map, but only provides a way to identify progenitors. The fact that these are two different concepts becomes quite evident with another specific example: In the figure below, if we map the green triangles (old mesh) obtained by a fate map (on the left) into the old mesh of the backward map (on the right), we will find that the possible progenitor region identified is much broader than the original labeled region from which the fate map originated – 3 green triangles instead of 1.

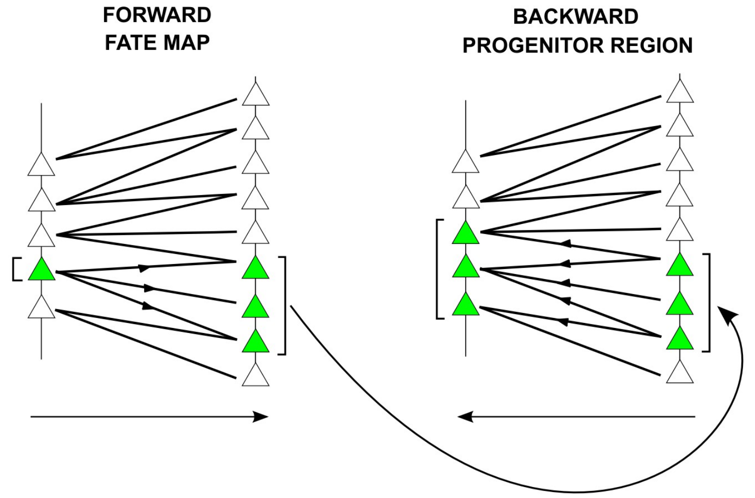

In addition the tissue movement map can also be read forward and backward considering additional levels of diffusion / cell mixing:

By reading it forward with additional diffusion (*lower left-hand panel*) fate maps can be computed as explained above but considering a higher degree of mixing between the cells. In the specific example (which represents only one of the 72 steps of mesh interpolation) the diffusion allows the dye values to spread into neighbouring triangles, during the 1 hour period when the younger mesh is being stretched, before the interpolation is performed. By diffusing the dyes before the interpolation a greater mix between fates is obtained. Similarly the same procedure can be performed in reverse (*lower right-hand panel).*

As an extra control for the conclusions made in Figure 9, we have also re-done the reverse simulation without diffusion – to shown that these results do not depend on the extra degree of diffusion considered previously. Below we show the results of this "triangle-mapping-only" version of the reverse map (bottom row), in comparison to the previous version from Figure 9 (top row). Although the sizes of progenitor overlap regions are slightly smaller, they are still clearly evident, supporting our general conclusion that early specification of all PD zones is not an accurate description of limb bud development.

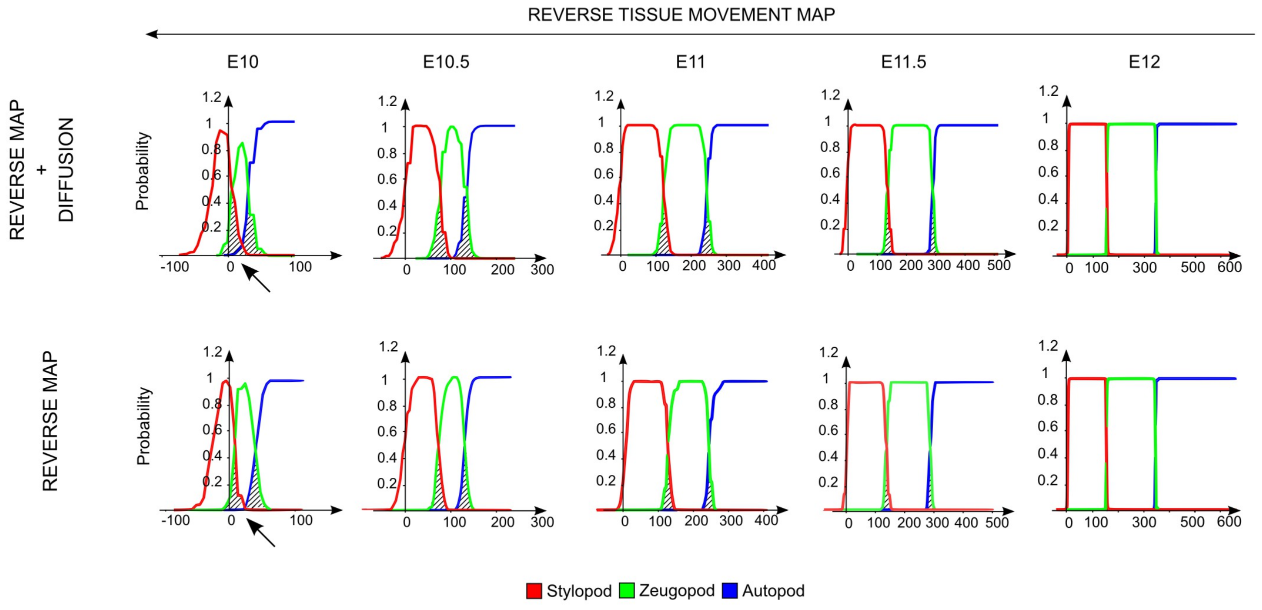

A considerable degree of mixing is observed also in the reverse map without diffusion. The black arrows highlight the major differences between the two reverse maps at E10.
